# Binding of TFIIIC to SINE Elements Controls the Relocation of Activity-Dependent Neuronal Genes to Transcription Factories

**DOI:** 10.1371/journal.pgen.1003699

**Published:** 2013-08-15

**Authors:** Luca Crepaldi, Cristina Policarpi, Alessandro Coatti, William T. Sherlock, Bart C. Jongbloets, Thomas A. Down, Antonella Riccio

**Affiliations:** 1 MRC Laboratory for Molecular and Cell Biology, University College London, London, United Kingdom; 2 Wellcome Trust Gurdon Institute, University of Cambridge, Cambridge, United Kingdom; 3 Department of Neuroscience, Physiology and Pharmacology, University College London, London, United Kingdom; Stanford University School of Medicine, United States of America

## Abstract

In neurons, the timely and accurate expression of genes in response to synaptic activity relies on the interplay between epigenetic modifications of histones, recruitment of regulatory proteins to chromatin and changes to nuclear structure. To identify genes and regulatory elements responsive to synaptic activation *in vivo*, we performed a genome-wide ChIPseq analysis of acetylated histone H3 using somatosensory cortex of mice exposed to novel enriched environmental (NEE) conditions. We discovered that Short Interspersed Elements (SINEs) located distal to promoters of activity-dependent genes became acetylated following exposure to NEE and were bound by the general transcription factor TFIIIC. Importantly, under depolarizing conditions, inducible genes relocated to transcription factories (TFs), and this event was controlled by TFIIIC. Silencing of the TFIIIC subunit Gtf3c5 in non-stimulated neurons induced uncontrolled relocation to TFs and transcription of activity-dependent genes. Remarkably, in cortical neurons, silencing of Gtf3c5 mimicked the effects of chronic depolarization, inducing a dramatic increase of both dendritic length and branching. These findings reveal a novel and essential regulatory function of both SINEs and TFIIIC in mediating gene relocation and transcription. They also suggest that TFIIIC may regulate the rearrangement of nuclear architecture, allowing the coordinated expression of activity-dependent neuronal genes.

## Author Summary

In neurons, acetylation of histones and other epigenetic modifications influence gene expression in response to synaptic activity. Genes that are concomitantly expressed in response to stimulation are transcribed at specific nuclear foci, known as transcription factories (TFs) that are enriched with active RNA Polymerase II and often include specific transcription factors. Here, we show a novel regulatory role for Short Interspersed Elements (SINEs) located in the proximity of activity-regulated genes. SINEs represent a new class of regulatory sequences that function as coordinators of depolarization-dependent transcription. Binding of the general transcription factor TFIIIC to SINEs regulates activity-dependent transcription, relocation of inducible genes to transcription factories and dendritogenesis. Our study provides new fundamental insights into the mechanisms by which relocation of inducible genes to transcription factories and changes of nuclear architecture coordinate the transcriptional program in response to neuronal activity.

## Introduction

The adaptation of living organisms to their surroundings depends on their ability to fine-tune their behaviors in response to novel conditions. Failure to adapt rapidly to environmental changes compromises behavioral responses such as memory formation, which are essential for survival. At a cellular level, exposure to environmental enrichment correlates with a number of morphological changes, ranging from increased dendritic growth and branching to enhanced synaptogenesis and hippocampal neurogenesis [1]. Many of these enrichment-mediated cellular changes depend on the expression of specific genes involved in neuronal plasticity, such as the neurotrophins Brain Derived Neurotrophic Factor (BDNF) and Nerve Growth Factor (NGF), as well as synaptic proteins, including PSD95 and glutamate receptor subunits [2]–[4].

In neurons, transcriptional activation depends on a host of molecular events that act on at least three distinct, yet interconnected levels. The best-characterized mechanism relies on the interaction of nuclear factors with specific regulatory elements within gene promoters. Binding of co-repressor complexes often containing histone deacetylases, inhibits transcription by inducing chromatin modifications that prevent the recruitment and assembly of RNA polymerase II (RNAPII) complexes [5]. Conversely, interaction of co-activators with specific DNA sequences within gene promoters is associated with stimulus-dependent transcription [6]. Transcriptional activators and repressors are often simultaneously detected on promoters of both active and inactive genes [7], suggesting that a dynamic balance between gene activation and inhibition may determine the transcriptional outcome.

A second level of regulation relies on epigenetic modifications of histone proteins, including acetylation, methylation and phosphorylation. Post-translational modifications of histones and DNA methylation induce structural changes of the chromatin, and provide docking sites for the recruitment of transcriptional cofactors [6], [8], [9]. Importantly, chromatin modifications contribute to determining whether genes are silenced, expressed or maintained transcriptionally inactive, yet poised for activation. Promoters of poised genes are characterized by stalled RNAPII [10], [11] and although they share several epigenetic marks with actively transcribed genes, they become expressed only upon stimulation [12], [13]. This mechanism is especially relevant for genes that undergo very rapid stimulus-dependent transcriptional activation, such as immediate-early genes (IEGs) [14]. Both binding of transcription factors and epigenetic modifications associated with transcriptional activation are also observed at extragenic regions that function as enhancers [15], [16].

Finally a third level of transcriptional regulation depends on the three-dimensional organization and functional compartmentalization of the nucleus [17]–[19]. In several mammalian cell types, transcription of genes that are concomitantly activated in response to extracellular stimuli or during cell differentiation occurs specifically at intranuclear foci enriched with active RNAPII [20], [21]. These transcriptional hubs are known as transcription factories (TFs) and in addition to RNAPII, they often include transcription factors [19], [22].

To investigate how synaptic activity regulates gene transcription *in vivo*, we performed a genome-wide ChIPseq analysis of histone H3 acetylation at lysine residues 9 and 14 (H3K9K14ac) using somatosensory cortex of mice exposed to novel enriched environmental (NEE) conditions. *De novo* acetylated regions contained Short Interspersed Elements (SINEs) that interacted with the general transcription factor TFIIIC. SINEs are generated by retrotransposition of genes transcribed by RNA polymerase III (RNAPIII) and, although they are present within the genome in hundreds of thousands of copies, their function is only partially understood [23]. TFIIIC is part of the RNAPIII complex and mediates both the recognition and the binding of RNAPIII to gene promoters [24]. Interestingly, in mammalian cells, ChIPseq analyses of TFIIIC occupation demonstrated a widespread binding of TFIIIC across the genome in the absence of RNAPIII [25]–[27], suggesting that its role is not limited to mediating RNAPIII-dependent transcription.

Here, we show that in neurons, TFIIIC regulates activity-dependent relocation to TFs and transcription of RNAPII-dependent gene loci. Inhibition of TFIIIC levels dramatically increased both dendritic growth and branching. The eukaryote genome harbours hundreds of TFIIIC binding sites with unknown function. Our findings indicate that, in neurons, TFIIIC mediates the rearrangement of nuclear architecture, possibly by coordinating the simultaneous expression of activity-dependent neuronal genes necessary for dendritic growth.

## Results

### Most genes induced by NEE are poised for transcription

Exposure to NEE represents a physiological mode of neuronal stimulation that combines sensory, cognitive and social stimuli [1], [28]. In rodents, NEE elicits a variety of plastic responses, including increased dendritic arborization, synaptic density, neurogenesis, and improved memory functions. Moreover, in humans, exposure to an enriched environment has a beneficial effect on many pathological conditions, contributing to ameliorate symptoms associated with several neurodegenerative disorders [1], [29]. The mechanisms underlying these changes are not yet fully elucidated, however enhanced synaptic activity and neurotrophin signaling are implicated [29].

We first studied whether neuronal activation controls gene expression *in vivo* by exposing adult mice to NEE for 45 minutes (Figure S1A). As a control, mice were maintained in standard cages for the same amount of time. A short-term exposure was chosen in order to characterise the early transcriptional events that regulate activity-dependent neuronal functions [30]. In situ hybridization of mouse somatosensory cortices demonstrated that a short-term exposure to NEE was sufficient to elicit a robust expression of many activity-dependent genes, including *c-Fos* [31] and *Arc* [32], [33] both in the cortex and hippocampus (Figure S1B–C).

To characterize the epigenetic modifications associated with the transcriptional program activated by NEE, we employed ChIPseq assay, a technique that combines chromatin immunoprecipitation with large-scale direct ultrahigh-throughput DNA sequencing. Various posttranslational modifications of histones have been associated with “open” chromatin and gene expression, including histone H3 acetylation at lysine residues 9 and 14, and histone H3 methylation on lysine 4. H3K9K14ac represents an epigenetic mark, which is particularly useful in the identification of active neuronal genes, as this chromatin modification has been detected on both promoters and extragenic enhancers of actively transcribed genes in cortical neurons [15], [34]. Mice were either exposed to NEE conditions or maintained in standard cages, the somatosensory cortex was rapidly dissected and regions differentially acetylated on H3K9K14 in response to synaptic activity were analysed by ChIPseq. At least 3.5 millions unique reads were obtained from control and NEE-exposed ChIPseq libraries (Table S1). Differentially acetylated genomic regions were identified by counting all reads mapping within 2 kb sliding windows across the genome and the differential enrichment relative to a Binomial null model was analysed (see the Materials and Methods section for details). This approach provided statistical robustness despite the relatively low number of sequenced reads. 9,811 regions ranging from 2 to 8.5 kbp in size showed increased levels of H3K9K14ac (referred here as +Δac), whereas 13,158 regions, ranging from 2 to 11.5 kbp had decreased H3K9K14ac (−Δac; detailed results of the ChiPseq screens are provided in Dataset S1). Most regions that presented activity-dependent changes of H3K9K14ac levels encompassed the body of annotated genes (Figure S1D) whereas 895 +Δac and 1,263 −Δac regions overlapped with transcription start sites (TSS).

Next, we analysed the transcriptional response to NEE in the somatosensory cortex of mice that were exposed to enriched environmental conditions for 3 hours or maintained in standard cages. Genome-wide microarray analysis revealed that 11,071 genes were expressed in both control and NEE-exposed mice (henceforth referred to as Constitutively Expressed, CE) while 17,451 remained transcriptionally silent (Constitutively Silent, CS). Among the transcripts that were differentially expressed, 196 were induced by at least 1.24 fold in response to NEE (NEE-induced, NI) whereas 70 were repressed (NEE-repressed, NR; Table S2). Transcriptional activation of *Arc, c-Fos* and *Gadd45b* in response to NEE was further validated by qRT-PCR analysis (Figure S1C). Surprisingly, among the 1,663 genes showing NEE-dependent increase of H3K9K14ac at the TSS, only 28 were also transcriptionally activated (Figure S1E). A possible explanation for this finding is that a more prolonged stimulation may be necessary in order to trigger the transcription of most *de novo* acetylated genes.

**Figure 1:**
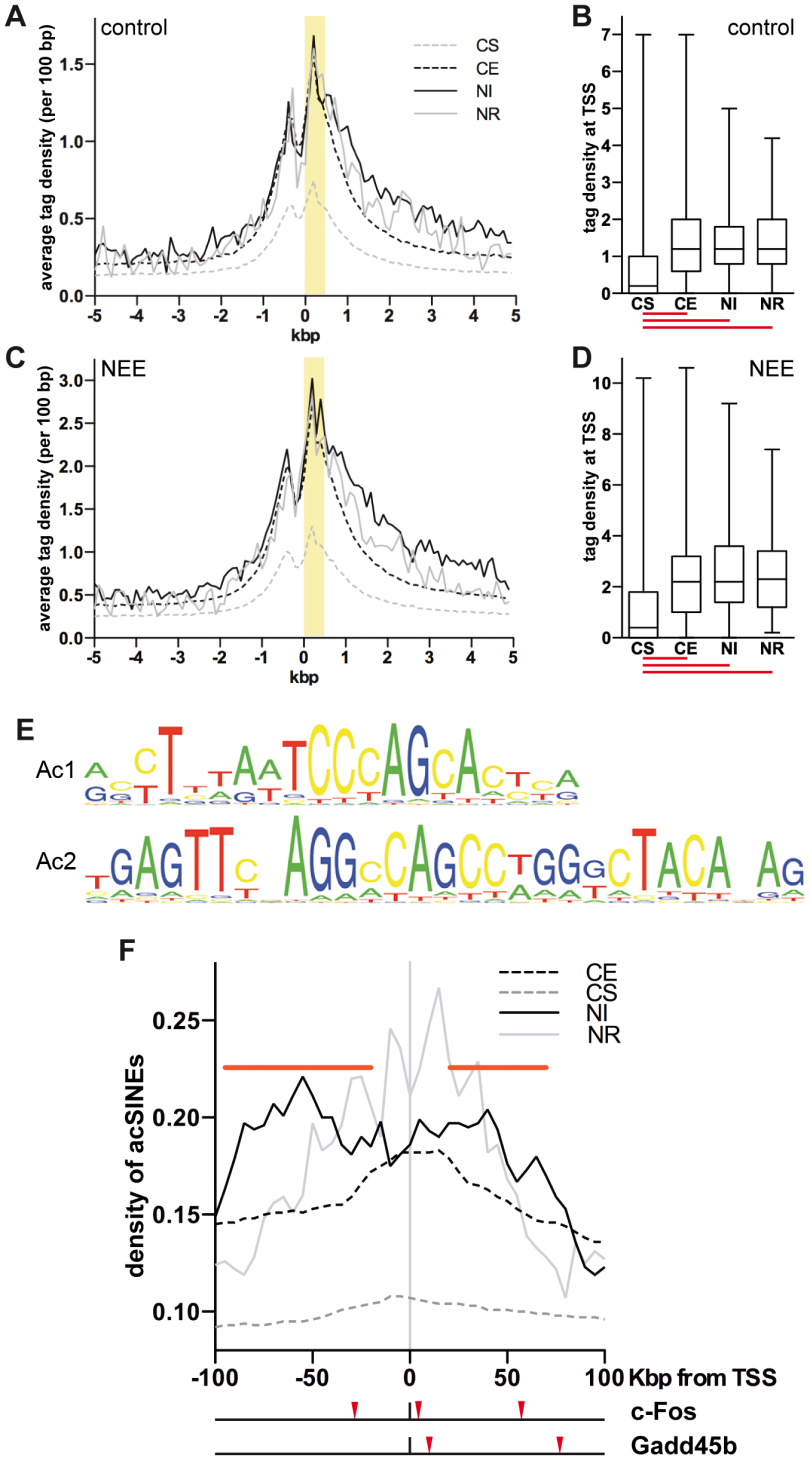
Acetylated SINEs are a distinctive feature of NEE-induced genes. (A and C) Average H3K9K14ac ChIPseq tag density of constitutively silent (CS, gray dashed line), expressed (CE, black dashed line), NEE-induced (NI, black solid line) and repressed (NR, gray solid line) genes in somatosensory cortex of control mice (A) and mice exposed to NEE for 45 minutes (C). For each gene group, the average density of ChIPseq reads per 100 bp window is plotted relative to the TSS. (B and D) Box and whiskers plots summarizing the distribution of ChIPseq tag density at +0/+0.5 kbp from TSS, highlighted in yellow in (A) and (C), for each of the four gene groups, in control (B) and NEE-stimulated (D) mouse cortex. Lower and upper whiskers indicate the minimum and maximum value of the distribution, respectively. The lower and upper limits of the box indicate the 25^th^ and 75^th^ percentile, respectively. The solid line denotes the median. The red lines below each box plot indicate whether the difference between the medians of two data sets is statistically significant (P<0.0001, two-tailed Mann-Whitney test). The tag density values for control (A, B) and NEE-stimulated (C, D) cortex are not directly comparable, as they have not been normalized across samples. (E) Ac1 and Ac2 motifs were found by applying motif inference on +Δac regions. (F) Average density of *de novo* acetylated SINEs distribution for constitutively silent (CS, gray dashed line), constitutively expressed (CE, black dashed line), NEE-induced (NI, black solid line) or NEE-repressed (NE, gray solid line) genes. For each gene group, the average density of *de novo* acetylated SINEs per 50 kbp window (at 5 kbp intervals) is plotted. Horizontal red bars represent regions where the SINE density of NI genes is significantly higher than that of CE genes (P<0.05, two-tailed Mann-Whitney test). Red arrowheads under the graph indicate the *de novo* acetylated SINEs detected within 100 kbp of the *c-Fos* and *Gadd45b* TSSs.

To further characterize the correlation between gene promoters acetylation and transcription, H3K9K14ac profiles at TSSs of constitutive (CE), silent (CS), NEE-induced (NI) and NEE-repressed (NR) genes were analyzed before and after NEE exposure (Figure 1A–D and Table S2). In control conditions, H3K9K14ac tag density analysis showed an enrichment of at least 2-fold at CE gene promoters, when compared to CS (Figure 1A,B). H3K9K14ac profiles of both NI and NR gene pools almost perfectly overlapped with CE genes, indicating that acetylation levels of promoters undergoing rapid changes of transcriptional activity are remarkably stable. Surprisingly, promoters of NI genes were already acetylated prior to exposure to NEE (Figure 1A,B), suggesting that, in unstimulated somatosensory cortex, promoters of activity-dependent genes are hyperacetylated and poised for transcription. A recent study has shown that in neurons maintained in resting conditions, the binding of RNAPII to promoters of many activity-dependent IEGs was comparable to depolarized neurons. Interestingly, RNAPII stalling was necessary for fast transcriptional activation of IEGs [14]. It is therefore conceivable that in unstimulated neurons, the combination of promoter acetylation on H3K9K14 and RNAPII binding represents a feature of rapidly inducible genes.

### Acetylated SINEs are enriched in the proximity of NEE-induced genes

The finding that promoters of both stably expressed and inducible genes shared a virtually indistinguishable pattern of H3K9K14ac, prompted us to investigate whether acetylation of chromatin regions other than TSSs were uniquely associated with NEE-activated genes. To identify putative regulatory elements that mediated transcriptional activation of inducible genes, we employed the motif-prediction tool NestedMICA [35] and analysed the +Δac regions identified by ChIPseq in the cortex of mice exposed to NEE. Motif inference identified two elements of 20 and 29 bp (referred here as Ac1 and Ac2) that were over-represented in +Δac regions (Figure 1E). Further investigation revealed that both motifs overlapped with distinct portions of B1 SINEs (Figure S1F). SINEs, together with the Long Interspersed Elements (LINEs) and the Long Terminal Repeats (LTRs) are among the most abundant classes of retrotransposons present in the mammalian genome. LINE-mediated retrotransposition has been demonstrated to occur at a much higher rate than previously estimated, thereby contributing to generate cell diversity. In neurons, L1 retrotransposition alters the expression of many neuronal genes, influencing cell fate *in vitro* and inducing somatic mosaicism *in vivo* [36], [37]. Because SINEs are short elements with no coding capacity, they show hardly any retrotransposition activity and have long been considered as “junk DNA”. However, a number of studies demonstrated that they possess diverse and evolutionarily important biological functions, from the regulation of transcription to the targeting of mRNAs [38]. Recent studies have also demonstrated that SINEs may influence the development of the nervous system. AmnSINEs, a highly conserved family of SINEs identified in the genome of amniota, possess enhancer properties and contribute to the expression of genes necessary for brain development. For example, amnSINEs proximal to *Fgf8* and *Satb2* genes, possibly acting as tissue-specific enhancers of transcription, recapitulate the expression pattern of *Fgf8* and *Satb2* in the developing forebrain [39], [40].

SINEs are characterised by an RNAPIII promoter encoding two elements known as A and B boxes, although within SINE families, variable levels of conservation are observed [24], [41]. The motif Ac2 that we identified includes 15 bp of the 16 bp long consensus sequence of the B box (Figure S1F). Despite the fact that Ac1 and Ac2 elements specifically identified B1 SINEs, the occurrence of all SINE families within +Δac regions was also determined. Alu (or B1), B4 and MIR SINEs displayed a significantly higher frequency within +Δac regions when compared with randomly selected genomic sequences of comparable size (Figure S1G). Conversely LINEs showed a lower frequency within +Δac regions. At least one SINE was present within 73% of 9,811 +Δac regions. Given that many sequenced tags derived from repetitive elements (such as SINEs) cannot be univocally mapped and were therefore excluded from the ChIPseq analysis, it is likely that these data underestimate the frequency of SINEs within +Δac regions. Because +Δac regions are located mostly within gene loci (Figure S1D), and gene-rich regions are characterized by a higher presence of SINEs [42], the increased SINEs frequency in +Δac regions shown in Figure S1G may simply reflect the genomic context of the acetylation changes. To exclude this possibility, the relative distribution of *de novo* acetylated SINEs within the genomic context was analyzed using genes grouped accordingly to their transcriptional response to NEE. Despite having stable and comparable levels of acetylation at the TSS (Figure 1A–D), NI genes showed a higher frequency of acetylated SINEs both upstream (−95/−20 kbp) and downstream (+20/+75 kbp) of their TSSs (Figure 1F), when compared to housekeeping (CE) genes. No significant difference in the distribution of acetylated SINEs was observed in NR genes, when compared to CE genes (Figure 1F). Due to the relatively low number of NR genes (70) the profile of acetylated SINEs is considerably scattered and does not allow a robust statistical analysis.

The analysis of the genomic regions surrounding the TSSs of NEE-induced genes revealed that although acetylated SINEs were significantly enriched at a distance greater than 20 kbp from the TSS (Figure 1F), in many cases, they were also found between −20 and +20 kbp from the TSS (Table 1). It should be noted that additional acetylated SINEs were discovered between 20 and 100 kbp from the TSS for all genes listed in Table 1 (Figure 1F and LC and AR, unpublished results). Our findings indicate that *de novo* acetylation of SINEs represents a landmark of inducible genes, suggesting that these elements may play a critical role in regulating activity-dependent transcription.

**Table 1:** Acetylated SINEs proximal to TSSs of NEE activated genes.

gene	fold induction (3 h NEE)	nearest acetylated SINE (with putative B box)	distance to TSS (kbp)	score (B box prediction)
FOS	3.88	RSINE1	3.9	++
SGK1	2.65	B1F	17.3	+
JUNB	2.06	B2_Mm1t	6.6	++++
NPAS4	2.04	B3	−1.6	+++
PPP1R3G	1.93	B1_Mur4	−1.3	+
PGLYRP1	1.69	B3	−10.3	++
HSPA1A	1.68	B2_Mm1a	10.4	++++
STK40	1.62	B1_Mus1	−2.6	+++
BAIAP2	1.60	B2_Mm1t	7.2	++++
HEXIM1	1.55	B2_Mm2	7.9	++++
GADD45B	1.55	B1F	9.4	++
SERPINH1	1.52	PB1D10	15.0	++
MAT2A	1.49	PB1D7	9.3	++
FMNL1	1.45	B1_Mus1	5.4	+++
DOK3	1.45	B2_Mm1a	9.5	+++
JMJD6	1.44	B1_Mm	−5.3	++
MKKS	1.44	ID4	4.8	++
DYRK3	1.44	ID4	5.7	+
1700123O20RIK	1.44	B2_Mm2	−7.8	+++
SFRS7	1.40	B1_Mur4	3.9	++
PDIA4	1.38	B1_Mur4	2.8	+++
ARID5B	1.37	B4A	23.0	++

### SINEs located near NEE-induced genes contain TFIIIC binding sites

**Figure 2:**
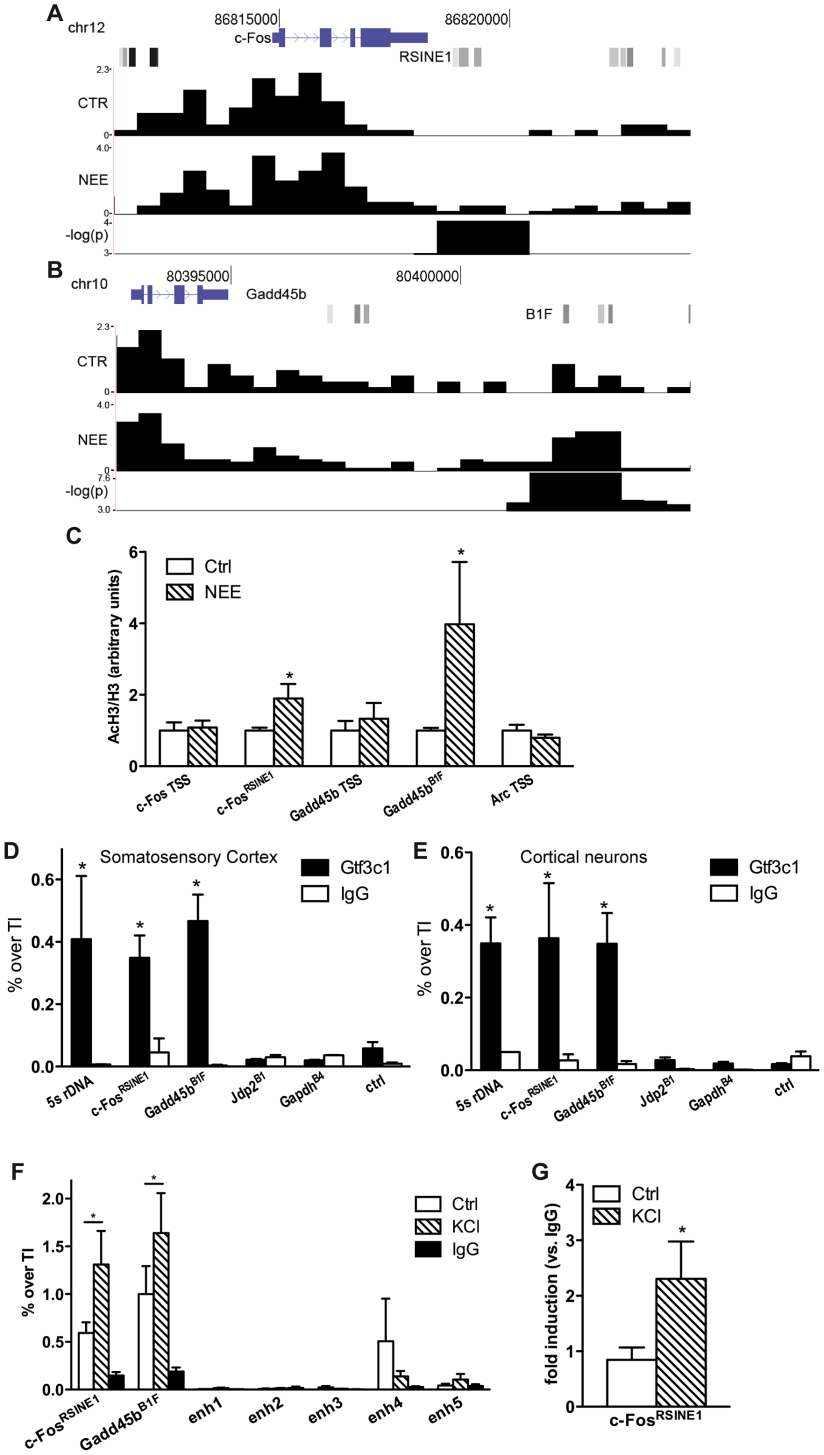
TFIIIC binds to *de novo* acetylated SINEs near inducible genes. (A and B) Changes in H3K9K14ac at *c-Fos* (A) and *Gadd45b* (B) loci. From the top, gene locus structure (blue), SINEs (shades of gray) with *c-Fos* ^RSINE1^ (A) and *Gadd45b* ^B1F^ (B) highlighted, H3K9K14ac tag density per 100 bp in control conditions (CTR) and in response to NEE (NEE), and −log(p-value) of regions of statistical difference in H3K9K14ac levels are shown. Tag density profiles are not directly comparable, as they have not been normalized across samples. (C) Exposure to NEE increased histone H3 acetylation at *c-Fos* ^RSINE1^ and *Gadd45b* ^B1F^ and not at TSSs. Adult mice were either exposed to NEE for 45 min or left untreated, somatosensory cortex was dissected and subjected to ChIP using either H3K9K14ac or histone H3 antibodies, followed by qPCR. Histograms show the ratio of immunoprecipitation efficiency between H3K9K14ac and H3 antibodies relative to total chromatin input (average and s.e.m. of at least 6 mice per condition are shown; *, P<0.05, Student's t-test). (D and E) Mouse somatosensory cortex (D) and primary cortical neurons (E) were subjected to Gtf3c1 ChIP followed by qPCR targeting *c-Fos* ^RSINE1^ and *Gadd45b* ^B1F^. Histograms show the efficiency of immunoprecipitation relative to total chromatin input, expressed as percentage of total input. 5S rRNA gene locus was used as positive control. *Jdp2* ^B1^, *Gapdh* ^B4^ and a genomic region devoid of SINEs and H3K9K14ac ChIPseq tags (ctrl) were used as negative control (average and s.e.m. of at least 3 experiments are shown; *, P<0.05, Student's t-test). (F) Gtf3c1 binding at *c-Fos* ^RSINE1^ and *Gadd45b* ^B1F^ increased in response to depolarization. Mouse primary cortical neurons were either exposed to 50 mM KCl for 45 min or left untreated, and subjected to ChIP using Gtf3c1 antibodies, followed by qPCR. Histograms show the immunoprecipitation efficiency of Gtf3c1 and control IgG antibodies relative to total chromatin input. Five previously identified *c-Fos* enhancers [16] showed no significant Gtf3c1 binding (average and s.e.m. of 3 experiments are shown; *, P<0.05, Student's t-test). (G) p300 was recruited to *c-Fos* ^RSINE1^ in response to depolarization. Mouse primary cortical neurons were either exposed to 50 mM KCl for 45 min or left untreated, and subjected to ChIP using p300 antibodies, followed by qPCR. Histograms show the immunoprecipitation efficiency of Gtf3c1 antibodies relative to control IgG (average and s.e.m. of 3 experiments are shown; *, P<0.05, Student's t-test).

In neurons, regulatory sequences with enhancer functions are found within a wide range of distances from the TSS. The *c-Fos* gene for example, contains at least five enhancers located between −40 and +15 kbp from the TSS [16]. Neuronal enhancers are characterized by both the presence of specific epigenetic modifications, including H3K4Me1, and by the binding to specific nuclear factors, such as CREB, SRF, Npas4 and CBP. To investigate whether acetylated SINEs may represent a new class of regulatory elements that control synaptic activity-dependent transcription in neurons, we analysed *c-Fos* and *Gadd45b*, two genes that fulfilled several important criteria: First, they are both activity-dependent genes with well-known functions in the nervous system [31], [43]; Second, following 45 minutes of exposure to NEE, both genes undergo rapid and robust transcriptional activation in the cortex (6.0±0.8 and 2.7±0.7 fold induction, respectively, as assessed by qRT-PCR (Figure S1C)); Third, ChIPseq analysis of the genomic regions surrounding the *c-Fos* and *Gadd45b* TSSs using somatosensory cortices of mice maintained in resting conditions or exposed to NEE revealed a stable acetylation profile, with the relevant exception of two regions encompassing RSINE1 and B1F, two SINEs located 3.9 and 9.2 kbp downstream from the *c-Fos* and *Gadd45b* TSSs, respectively (Figure 2A,B and S1H). It should be noted that additional *de novo* acetylated SINEs are present within 100 kbp from the TSS of both *c-Fos* and *Gadd45b* (Figure 1F). ChIP experiments confirmed that in somatosensory cortex, both *c-Fos* ^RSINE1^ and *Gadd45b* ^B1F^ underwent *de novo* H3K9K14 acetylation in response to NEE, while the acetylation levels of promoter regions remained unchanged (Figure 2C). Similarly, *c-Fos* ^RSINE1^ and *Gadd45b* ^B1F^ became acetylated when cortical neurons were exposed to potassium chloride (50 mM, 45 minutes), a paradigm of stimulation that induces neuronal depolarization, calcium influx and gene transcription, that is commonly used to mimic synaptic activation *in vitro* (Figure S2) [16], [44]–[46].

Although the *de novo* acetylated SINEs did not present DNA elements that may mediate the binding of nuclear factors previously identified as interacting with enhancers [16], the SINEs B box represents the consensus sequence for the multisubunit general transcription factor TFIIIC (Figure S1H) [24]. TFIIIC is part of the RNAPIII complex, and is best characterised in the context of its role in mediating the recruitment of the transcriptional complex to the B box located within the promoters of RNAPIII- transcribed genes [24]. TFIIIC comprises six subunits, some of which have known functions. For example, Gtf3c1 mediates the binding to the B box [24], while Gtf3c2 and Gtf3c4 possess histone acetyltransferase activity (HAT) *in vitro* [47]. Importantly, TFIIIC may interact and recruit the HAT p300 to tRNA gene promoters [48]. Recent ChIP-chip analyses performed in fission yeast demonstrated that TFIIIC subunits are detected in the absence of RNAPIII at many genomic sequences known as Extra-TFIIIC sequences (ETCs) [49], [50]. In yeast, binding of TFIIIC to ETC sites is necessary for tethering distant loci to the nuclear periphery, thereby contributing to the maintenance of the three-dimensional structure of chromosomes [49], [51]. ChIPseq screens performed in both human and mouse cells also identified a large number of ETC sites that are frequently located proximal to RNAPII-dependent genes [25]–[27]. Moreover, in HeLa cells, at least 181 SINEs contain ETC sites [26].

We first performed an *in silico* analysis to verify whether +Δac regions contained putative ETCs. A consensus sequence for a “B box-like ETC” resembling the B box located at RNAPIII promoters, was recently identified with a ChIPseq screen targeting a subunit of TFIIIC [25]. We employed NestedMICA to determine whether the B box-like ETC motif was over-represented within *de novo* acetylated regions. +Δac regions showed a significant over-representation of the B box-like ETCs when compared either to a random set of genomic sequences (p = 0.0029) or to a random set of sequences overlapping gene TSSs (p = 0.0032). Because +Δac regions harbour both putative TFIIIC binding sites and acetylated SINEs, we asked whether TFIIIC complexes interacted with *de novo* acetylated SINEs located in the proximity of activity-dependent genes. Indeed, the position-weight matrix of the B box-like ETC [25] predicted a putative TFIIIC binding site at the *c-Fos* ^RSINE1^ and *Gadd45b* ^B1F^ elements located downstream of *c-Fos* and *Gadd45b* TSSs, respectively (Figure 2A,B, Figure S1H and Table 1). Further analysis of acetylated SINEs located within 20 kbp from the TSS of NI genes consistently identified putative binding sites for TFIIIC (Table 1). ChIP experiments on mouse somatosensory cortex and primary cortical neurons confirmed that the TFIIIC subunit Gtf3c1 binds to both *c-Fos* ^RSINE1^ and *Gadd45b* ^B1F^ at levels comparable to the 5s rRNA gene (Figure 2D,E). Conversely, Gtf3c1 binding was not detected at *Jdp2* ^B1^ or *Gapdh* ^B4^, two SINEs located 9 and 15 kbp from the TSS of a NR and a CE gene respectively, that lack a conserved B box and are located in genomic regions not associated with H3K9K14ac tags (Figure 2D,E; Genomic coordinates of SINEs analysed are listed in Table S3).

To study whether dynamic changes of TFIIIC binding may represent a common feature of activity-dependent regulatory elements, neurons were exposed to KCl or left untreated and Gtf3c1 binding was tested on *Gadd45b* ^B1F^ and *c-Fos* ^RSINE1^. As a control, five previously identified *c-Fos* enhancers were analysed [16]. We found that TFIIIC interaction with *Gadd45b* ^B1F^ and *c-Fos* ^RSINE1^ increased in response to depolarization. In contrast no significant binding of Gtf3c1 was observed on *c-Fos* enhancers (Figure 2F). Interestingly, both p300 and the neuronal enhancer-associated histone marker H3K4Me1 were dynamically regulated on *c-Fos* ^RSINE1^ in response to depolarization (Figure 2G, CP and AR, unpublished observations). Although CBP is recruited to *c-Fos* enhancers [16], it was not detected on *Gadd45b* ^B1F^ and *c-Fos* ^RSINE1^. These findings, together with the observation that *Gadd45b* ^B1F^ and *c-Fos* ^RSINE1^ are transcribed in response to depolarization (CP and AR, unpublished observations) suggest that acetylated SINEs may represent a novel class of regulatory elements that control the expression of activity-dependent genes in neurons.

### TFIIIC regulates activity-dependent transcription

**Figure 3:**
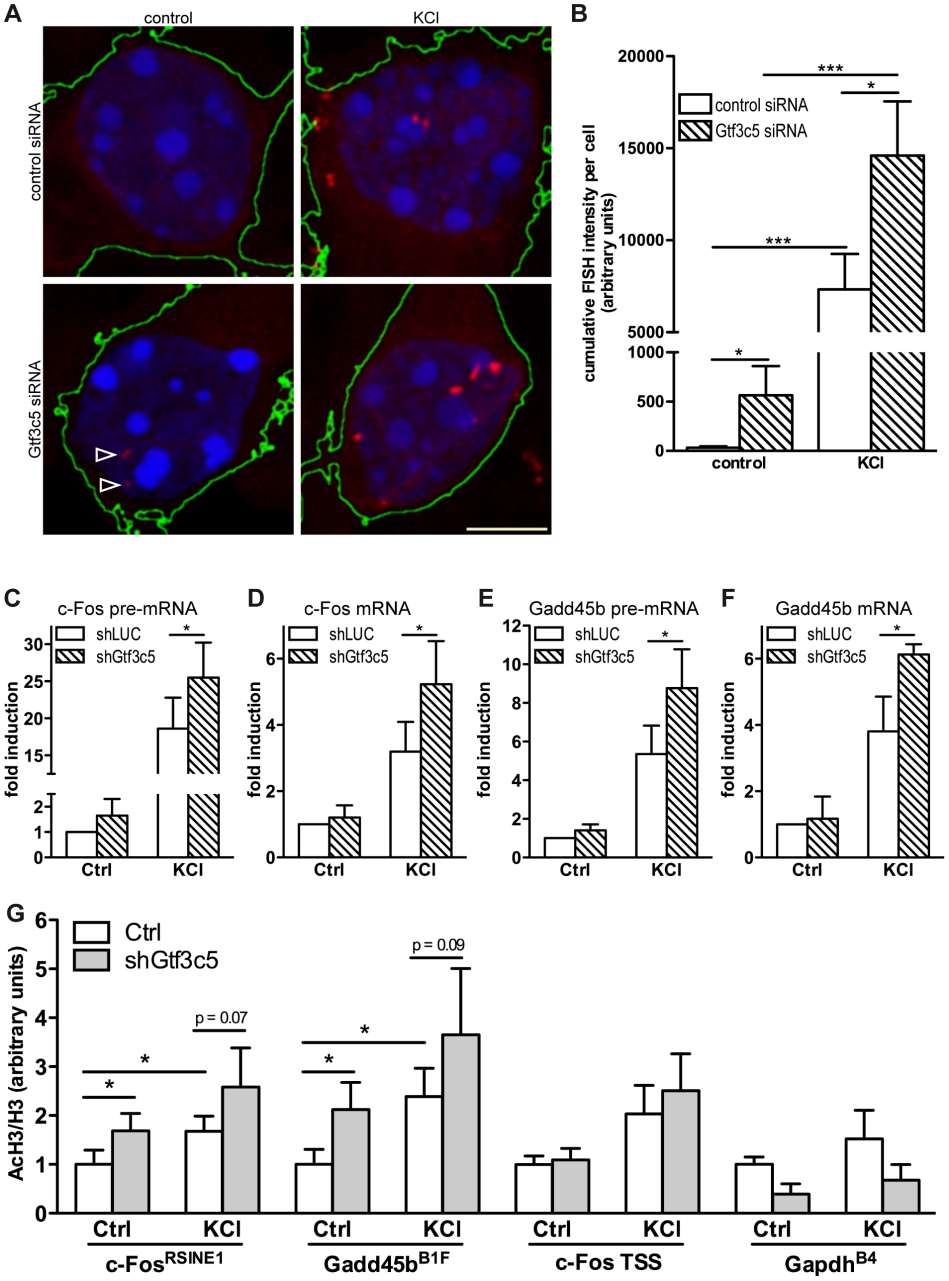
TFIIIC controls activity-dependent genes transcription and SINE acetylation. (A) Representative images of cortical neurons transfected with a GFP expression vector and either control or Gtf3c5 siRNA. Neurons were transfected at DIV2, after three days exposed to 50 mM KCl for 45 minutes or left untreated, and subjected to quantitative RNA-FISH analysis of c-Fos mRNA. Maximal z-projections of confocal stacks of transfected cells are shown. In GFP-expressing cells, *c-Fos* mRNA ribonucleoparticles (red) were detected by FISH (reconstructed cell edges are shown in green). Nuclei were stained with DAPI (blue). Ribonucleoparticles showing low levels of c-Fos mRNA are indicated by arrowheads. (B) Quantitative analysis of RNA-FISH experiments. For each neuron, total fluorescence intensity of all c-Fos mRNA particles was calculated. Average and s.e.m. of at least 25 cells per condition are shown (*, P<0.05; **, P<0.01; ***, P<0.001, two-way ANOVA). (C, D, E and F) Mouse primary cortical neurons were infected with lentiviral particles driving the expression of a short hairpin RNA targeting either firefly luciferase (shLUC, as a negative control) or Gtf3c5 (shGtf3c5). 4 days later cells were stimulated with either 50 mM KCl for 45 minutes or left untreated, then subjected to RNA extraction and cDNA synthesis, followed by qRT-PCR analysis of c-Fos and Gadd45b pre-mRNA (C, E) and mRNA (D, F). Silencing of Gtf3c5 was sufficient to drive a significant enhancement of activity-dependent transcription, as assessed both at the level of pre-mRNA (C, E) and fully processed RNA (D, F). (G) Primary cortical neurons were infected with lentiviral particles driving the expression of a short hairpin RNA targeting either firefly luciferase (shLUC, as a negative control) or Gtf3c5 (shGtf3c5). 4 days later cells were stimulated with either 50 mM KCl for 45 minutes or left untreated, then subjected to ChIP using either H3K9K14ac or histone H3 antibodies, followed by qPCR. Lentiviral-mediated silencing of Gtf3c5 enhanced histone H3 acetylation at *c-Fos* ^RSINE1^, *Gadd45b* ^B1F^ and not at *c-Fos* TSS and *Gapdh* ^B4^. Histograms show the ratio of immunoprecipitation efficiency between H3K9K14ac and H3 antibodies relative to total chromatin input (average and s.e.m. of 5 experiments are shown; *, P<0.05, Student's t-test).

The finding that TFIIIC interacted with SINEs that became acetylated in response to depolarization prompted us to investigate whether inhibition of TFIIIC influenced activity-dependent transcription. Mouse cortical neurons were transfected with either Gtf3c5 or control siRNA, depolarised with KCl and c-Fos mRNA levels were measured by quantitative RNA-FISH. The yeast homolog of Gtf3c5 stabilises the interaction of TFIIIC with the B box [52], although it does not directly bind to DNA. Transfected cells were identified by co-expression of a GFP vector. In non-stimulated neurons transfected with control siRNA, c-Fos transcripts were not detectable. In response to depolarization, discrete c-Fos mRNA ribonucleoparticles appeared in proximity to the nucleus (Figure 3A, upper panels and Figure S3A,B) and were clearly detectable in approximately 60% of stimulated neurons. This finding is in agreement with a recent report showing that depolarization of rat cortical neurons induced Arc expression only in about 50% of cells [14]. Similarly, in *Dictyostelium discoideum*, single cell analysis of IEGs transcription showed a high degree of variability between cells [53]. Quantitative fluorescence intensity analysis of c-Fos-containing ribonucleoparticles demonstrated that both in non-stimulated neurons and in response to depolarization, silencing of Gtf3c5 increased *c-Fos* transcription, when compared to control siRNA (Figure 3A,B). Interestingly, Gtf3c5 silencing increased fluorescence intensity rather than the number of cells containing detectable c-Fos signal (LC and AR, unpublished observations). In neurons transfected with Gtf3c5 siRNA, stimulus-independent increase of c-Fos expression was observed even in the presence of nifedipine, demonstrating that it was not due to depolarization events that may have been triggered by the silencing of Gtf3c5 (Figure S3C).

To investigate whether TFIIIC inhibition increased the expression of activity-dependent genes via transcriptional or posttranscriptional mechanisms, neurons were infected with lentiviral vectors driving the simultaneous expression of short hairpin RNA targeting either Gtf3c5 or firefly luciferase and GFP. Three days after infection, neurons were stimulated with KCl and subjected to qRT-PCR analysis. In neurons exposed to KCl, inhibition of Gtf3c5 was sufficient to enhance activity-dependent transcription of both pre-mRNA and fully processed mRNA of c-Fos (Figure 3C,D) and Gadd54b (Figure 3E,F). In contrast, the expression of 5s rRNA, a TFIIIC- and RNAPIII-dependent transcript, was not affected (Figure S3D). Although in neurons transfected with Gtf3c5 siRNA significant levels of c-Fos transcript were observed by RNA FISH in the absence of stimulation (Figure 3A,B), a similar increase was not detected by qRT-PCR, when neurons were infected with shGtf3c5. This discrepancy was probably due to the relatively low infection efficiency achieved in this set of experiments, which resulted in incomplete silencing of Gtf3c5 (Figure S3D). In contrast, single-cell RNA-FISH exclusively allows the analysis of transfected neurons, greatly improving the accuracy of mRNA quantification.

In agreement with the observation that TFIIIC inhibition disrupts the regulated transcription of activity-dependent genes, *c-Fos* ^RSINE1^ and *Gadd45b* ^B1F^ acetylation was higher in neurons infected with shGtf3c5 under both basal and stimulated conditions (Figure 3G). The increase of SINE acetylation was even more remarkable when infection efficiency of shGtf3c5 lentivirus and Gtf3c5 inhibition (both around 60%) were taken into account. Acetylation of *c-Fos* promoter and *Gapdh* ^B4^ was unchanged, indicating that inhibition of TFIIIC specifically affects regulatory SINEs (Figure 3G). It should be noted that expression levels of TFIIIC subunits were not changed in the cortices of mice exposed to NEE or in cortical neurons stimulated with KCl (Figure S3E, F). Moreover, KCl depolarization did not affect neuronal viability (Figure S3G). Taken together, these findings demonstrate that inhibition of TFIIIC induces unregulated transcription and enhances gene expression both in resting condition and in response to depolarization. Thus, TFIIIC may act as a “brake” of transcription that contributes to maintaining activity-regulated genes inhibited in the absence of stimulation.

### Neuronal genes relocate to transcription factories in response to depolarization

In yeast, binding of TFIIIC to ETCs regulates the three-dimensional arrangement of chromatin, resulting in tethering of chromosomal regions to discrete clusters at the nuclear periphery, where chromatin is maintained in a transcriptional repressed state [49], [51]. The eukaryote genome harbours several hundred ETCs of unknown function [25]–[27] that similarly to yeast may play a role in positioning genes within the nucleus, thereby regulating their expression. Visualization of transcription sites in mammalian nuclei has revealed that RNAPII complexes are distributed in discrete foci known as transcription factories (TFs) [19], [54]. Importantly, in mouse B lymphocytes, the relocation of genes to TFs mediates stimulus-dependent transcription [55].

**Figure 4:**
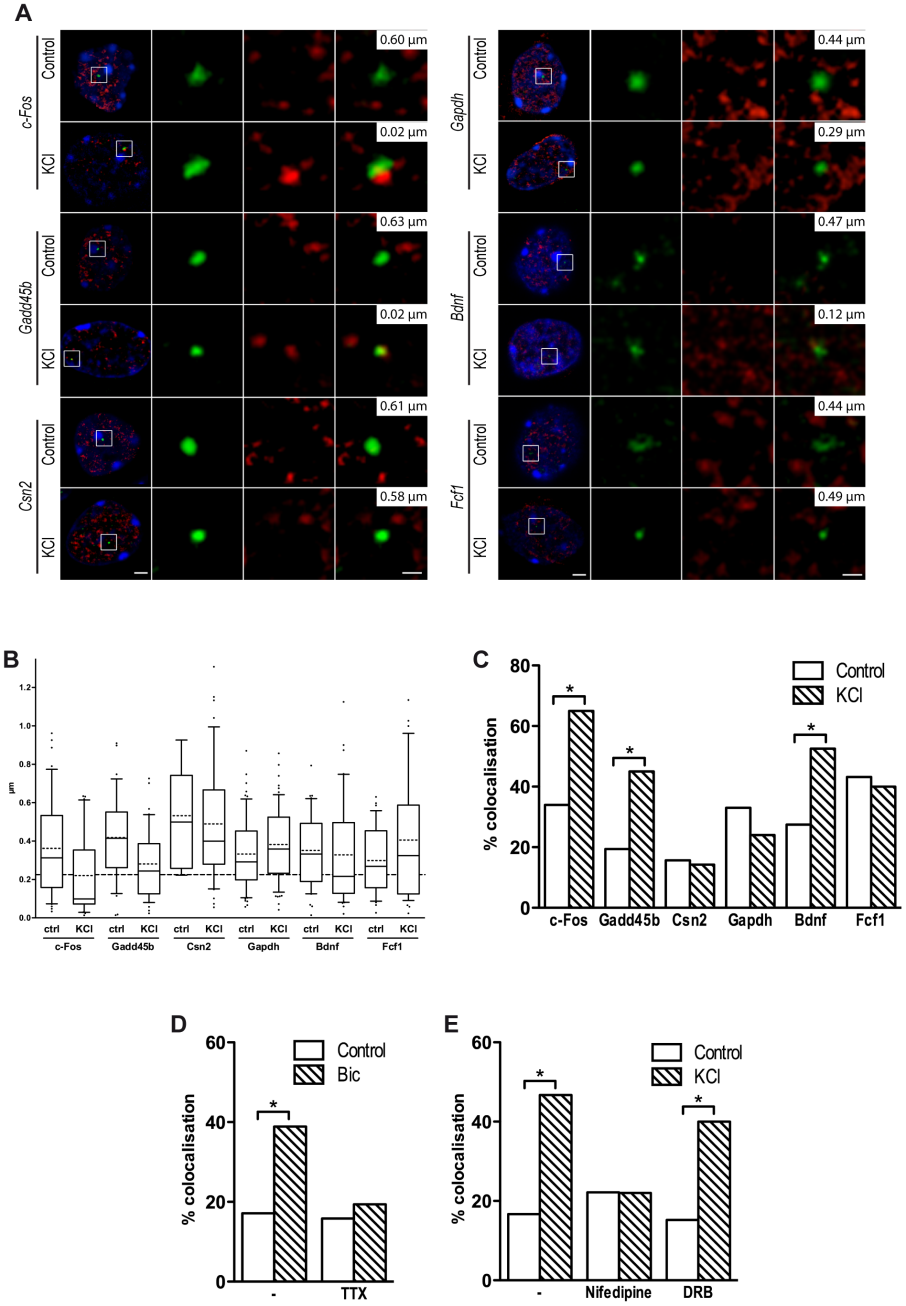
Inducible genes relocate to TFs in response to depolarization. (A) Representative images of single confocal sections of immuno-DNA FISH experiments showing nuclear localization of *c-Fos*, *Gadd45b*, *Csn2*, *Gapdh*, *Bdnf* and *Fcf1* loci (green) relative to TFs. TFs were detected by RNAPII-ser5P immunostaining (red), in cortical neurons either stimulated with 50 mM KCl for 45 minutes or left untreated. Nuclei were stained with DAPI (blue). For each image series, the distance between the centre of the FISH signal and the nearest TF is indicated (top right inset). Scale bars, 2 µm (images on the left) and 0.5 µm (magnified images). (B) Box and whiskers plot of the distribution of the distance between *c-Fos*, *Gadd45b*, *Csn2*, *Gapdh*, *Bdnf* and *Fcf1* loci and the nearest TF. Neurons were maintained either in basal conditions or exposed to 50 mM KCl for 45 minutes. Whiskers denote the 90th and 10th percentiles, box edges denote the 75th and 25th percentiles, solid lines denote medians, dashed lines denote averages. (C) Percentage of co-localization with TFs of *c-Fos*, *Gadd45b*, *Csn2*, *Gapdh*, *Bdnf* and *Fcf1* gene loci both in basal conditions an in response to KCl (*, P<0.05, Fisher's exact test). For each condition shown in (B) and (C), 31 to 64 FISH signals were analysed. (D) DIV10 cortical primary neurons were stimulated with 50 µM bicuculline for 45 minutes, either in the presence of 1 µM tetrodotoxin (TTX) or alone, and analysed by immuno-DNA FISH. Histograms show the percentage of co-localization with TFs of *c-Fos* gene locus (*, P<0.05, Fisher's exact test; n = 36 to 41 FISH signals per condition). (E) Cortical primary neurons were stimulated with 50 mM KCl for 45 minutes, either in the presence of 5 µM nifedipine, 50 µg/ml DRB or alone, and analysed by immuno-DNA FISH. Histograms show the percentage of co-localization with TFs of *c-Fos* gene locus (*, P<0.05, Fisher's exact test; n = 30 to 41 FISH signals per condition).

We first studied whether relocation of activity-regulated neuronal genes to TFs may be detected in nuclei of cortical neurons using immuno-DNA FISH. This technique allows the simultaneous detection of gene loci and RNAPII foci without affecting the three-dimensional structure of the nucleus (Figure 4A). Immunostaining of mouse cortical neurons with an antibody that recognizes a form of RNAPII associated with transcriptional initiation (RNAPII phospho-serine 5) [17], [54] detected 217±50 TFs (n = 27 cells). This finding is in agreement with the number of TFs observed in mouse fetal brain [54]. Computational analysis of the confocal image stacks was employed to quantify the degree of co-localization between gene loci and TFs. The distance between the centre of each DNA-FISH signal and the nearest TF was measured, and the threshold for co-localization was set at 225 nm. This was the distance at which two of the smallest detectable objects were considered overlapping. In the nuclei of cortical neurons exposed to KCl, co-localization of both *c-Fos* and *Gadd45b* loci with TFs increased dramatically (Figure 4A–C). Similar results were observed when DNA FISH experiments were performed using cortical neurons cultured for 10 days and exposed to bicuculline, an antagonist of GABA_A_ receptors that enhances glutamatergic transmission (Figure 4D and S4A). Bicuculline- and KCl-dependent relocation to TFs was completely abolished by blocking depolarization with tetrodotoxin (TTX) or nifedipine, respectively (Figure 4D, E and S4A,B). Importantly, relocation of *c-Fos* was also observed when neurons were treated with the transcription inhibitor 5,6-dichloro-1-beta-D-ribofuranosylbenzimidazole (DRB; Figure 4E and S4B), confirming that TFs are stable subnuclear structures that exist independently of active transcription both in fixed and in living cells [56], [57].

Consistent with the hypothesis that relocation to TFs is a necessary step for the expression of most, if not all, activity-regulated genes, and in agreement with a recent study [58], we found that *Bdnf*, an activity-dependent gene that was induced upon NEE, relocated to TFs in response to depolarization. Although the *Bdnf* gene does not have an acetylated SINE located within 100 kbp from its TSS, it is possible that SINEs located further from the promoter may result in looping of the chromatin that induces relocation of *Bdnf* to TFs. Indeed, several studies have shown that chromatin loops promoted by distal regulatory elements, situated several hundreds kbp away from the TSSs of their target genes, are capable of triggering the relocation of gene loci to active chromatin hubs [59]–[61]. Similarly, an acetylated SINE was detected 2 kbp upstream the TSS of *Fcf1*, a gene that did not relocate to TFs following depolarization and was not expressed in response to neuronal activity (Figure 4A–C). Thus, as for most regulatory elements, the distance of acetylated SINEs from TSS is not necessarily predictive of their regulatory function. As additional controls, we tested the loci of both *Csn2*, a gene that is not expressed in primary cortical neurons and *Gapdh*, a gene that is costitutively expressed, and found that they did not relocate to TFs in response to depolarization (Figure 4A–C). Thus, in neurons, as for other cell types [55], relocation of gene loci to TFs represents a necessary event that mediates activity-dependent transcriptional activation.

### TFIIIC regulates activity-dependent relocation to transcription factories

**Figure 5:**
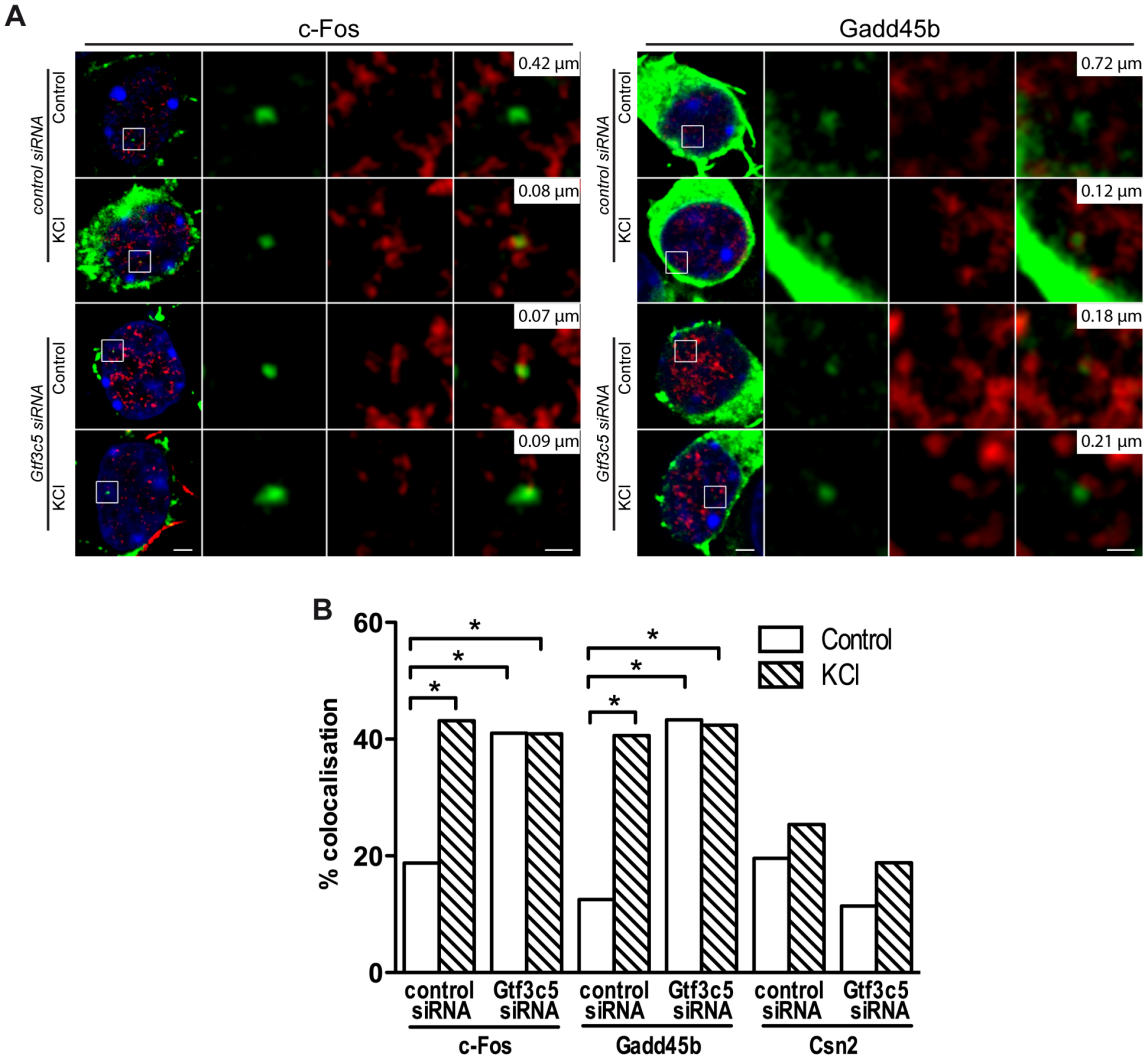
TFIIIC controls activity-dependent relocation of neuronal genes. Two days after plating, mouse primary cortical neurons were transfected with control or Gtf3c5 siRNA in combination with an eGFP-actin expression vector. After three days, neurons were stimulated with 50 mM KCl for 45 minutes or left untreated and analysed by immuno-DNA FISH targeting *c-Fos*, *Gadd45b* and *Csn2* loci. (A) Representative images of single confocal sections of immuno-DNA FISH experiments showing the nuclear localization of *c-Fos* and *Gadd45b* loci (green) relative to TFs, detected by RNAPII-ser5P immunostaining (red). GFP staining was confined outside the nuclear space (stained with DAPI, blue) and did not interfere with the FISH signal. For each image series, the distance between the centre of the FISH signal and the nearest TF is indicated (top right inset). Scale bars, 2 µm (images on the left) and 0.5 µm (magnified images). (B) Percentage of co-localization with TFs of *c-Fos*, *Gadd45b* and *Csn2* gene loci both in basal conditions an in response to KCl in neurons transfected with either control or Gtf3c5 siRNA (*, P<0.05, Fisher's exact test; n = 32 to 44 FISH signals per condition).

To study whether TFIIIC regulates the tethering of inducible gene loci to TFs, the Gtf3c5 subunit of TFIIIC was silenced by siRNA transfection (Figure S5A). Transfected neurons were identified by co-transfection of an expression vector encoding eGFP-tagged actin that remains confined in the cytoplasm, allowing the detection of fluorescein-labeled DNA-FISH probe within the nucleus (Figure 5A). In resting conditions, transfection of Gtf3c5 siRNA increased the co-localization of *c-Fos* and *Gadd45b* loci with TFs, at levels comparable to cells transfected with control siRNA and exposed to KCl (Figure 5A,B and S5B,C). Gtf3c5 silencing did not affect the co-localization of the silent gene *Csn2* (Figure 5B and S5D). Interestingly, in neurons transfected with Gtf3c5 siRNA, KCl treatment did not further increase relocation of *c-Fos* and *Gadd45b* loci to TFs (Figure 5B and S5B,C), indicating that knockdown of a TFIIIC subunit entirely mimicked the effects of neuronal depolarization on transcription. Enhanced relocation of *c-Fos* under basal conditions was also observed in neurons cultured in the presence of nifedipine, demonstrating that it was not dependent on depolarization events that may have been triggered by the silencing of Gtf3c5 (Figure S5E). The specificity of Gtf3c5 silencing was confirmed by generating a “rescue” siRNA-resistant form of Gtf3c5 (myc-Gtf3c5R; Figure S5F,G). Cortical neurons were transfected with expression vectors encoding myc-Gtf3c5R with shRNA targeting either Gtf3c5 (shGtf3c5) or firefly luciferase (shLuc, as a negative control) and subjected to immuno-DNA FISH. In resting neurons, expression of myc-Gtf3c5R completely rescued the effect of Gtf3c5 silencing on *c-Fos* relocation to TFs (Figure S5H). Our findings indicate that in the absence of stimulation, TFIIIC inhibits *c-Fos* and *Gadd45b* transcription by preventing their relocation to TFs. In response to depolarization, genes undergo nuclear repositioning to TFs and this event creates a molecular context highly permissive to transcription.

### TFIIIC functions regulate dendritogenesis

**Figure 6:**
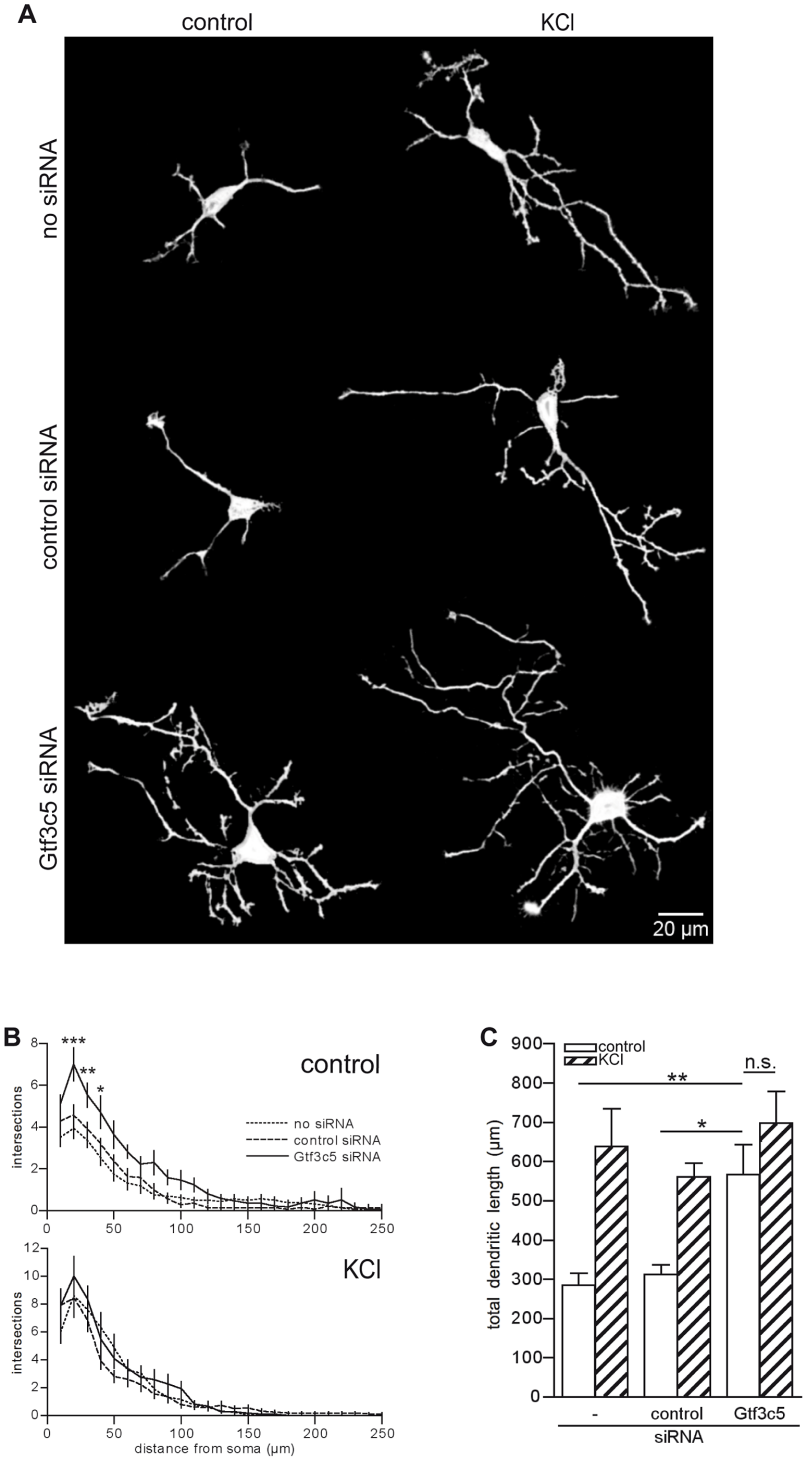
TFIIIC regulates dendritic growth. (A) Representative images of cortical neurons transfected with a GFP-expressing vector alone or in combination with either control or Gtf3c5 siRNA. Neurons were cultured for 2 days in basal conditions or in presence of 50 mM KCl, followed by GFP immunostaining. Shown are the enhanced profiles of neurons after reconstruction with a trainable segmentation tool. (B) Sholl profiles of neurons maintained in basal conditions (above) or exposed to KCl (below). For each distance point, the average number of intersections and s.e.m. are shown. At least 25 cells per condition were analysed (*, P<0.05; **, P<0.01; ***, P<0.001, two-way ANOVA). (C) Total length of the dendritic processes of the cells analysed in (B). Average and s.e.m. are shown (*, P<0.05; **, P<0.01, two-way ANOVA).

Neurons maintained in chronic depolarizing conditions undergo a dramatic increase of both dendritic length and branching [44], [45] and prolonged exposure to NEE ultimately leads to neuronal differentiation and dendritogenesis [1]. To investigate whether TFIIIC-dependent transcription influenced dendritic growth, mouse cortical neurons were transfected with an expression vector encoding GFP alone or in combination with control or Gtf3c5 siRNA. Neurons were left untreated or exposed to depolarizing conditions (50 mM KCl) for two days. Sholl analysis showed that in neurons exposed to KCl dendrites were more complex (Figure 6A,B and Figure S6) and total dendritic length was increased (Figure 6C). In contrast, neurons transfected with Gtf3c5 siRNA showed a dramatic increase of dendritogenesis when maintained in resting, non-depolarizing conditions. Remarkably, in neurons maintained in basal conditions, dendritic arborization and total dendritic length were comparable to neurons subjected to chronic KCl treatment that were either non-transfected or transfected with control siRNA (Figure 6). In neurons transfected with Gtf3c5 siRNA, depolarization also significantly enhanced dendritogenesis (Figure S6D). Interestingly, nifedipine failed to completely inhibit depolarization-dependent transcription of c-Fos in neurons transfected with Gtf3c5 siRNA (Figure S3C). Because Gtf3c5 silencing induced unregulated transcription of many genes that control neuronal functions, it is possible that as a result, these neurons presents both stronger synaptic contacts and more robust glutamatergic signaling, making them less sensitive to nifedipine. The growth and development of dendrites depends on *de novo* transcription of a number of genes, which are usually inactive in resting conditions [44]. Impairment of TFIIIC in non-stimulated neurons was sufficient to induce extensive dendritic growth comparable to chronic depolarization, indicating that upon Gtf3c5 silencing, many genes normally expressed only in response to depolarization underwent unregulated transcription.

## Discussion

Under physiological conditions the ever-changing environment constantly challenges the brain, which must respond with rapid adaptive strategies to ensure survival. For this to occur, it is essential that hundreds of activity-dependent genes become activated in an accurate and timely manner. Since the identification of *c-Fos* as the prototypical neuronal activity-dependent gene [31], the molecular mechanisms of transcriptional responses to depolarization have been extensively studied [8], [9], [62]. Following synaptic activity, increase of intracellular calcium levels triggers the initiation of signaling pathways that lead to the activation of transcription factors and their binding to specific gene promoters [30], [62], [63]. This event, together with the recruitment of histone modifying enzymes to chromatin, results in the rapid transcription of target genes [8], [9], [62]. In addition to promoters, many other regulatory sequences are scattered throughout the genome, with both transcriptional activating (enhancers) and repressing (silencers and insulators) functions [64]. In neurons, a novel class of enhancers associated with the p300 homolog CBP was recently shown to regulate activity-dependent gene expression [16]. Several models have been proposed to explain the molecular mechanisms by which enhancers activate gene expression, including chromatin looping that may favour both promoter-enhancer interaction and their relocation to transcriptionally active subnuclear compartments. We discovered that SINEs located in the proximity of inducible gene promoters represent a new class of regulatory elements that coordinate activity-dependent transcription. A genome wide ChIPseq screen performed using somatosensory cortex of mice exposed to NEE demonstrated that promoters of rapidly activated genes, including *c-Fos* and *Gadd45b* are remarkably stable and do not undergo changes of H3K9K14ac in response to synaptic activity. In contrast, SINEs located between −100 and +100 kbp from the TSS of inducible gene promoters became rapidly acetylated. Previous studies have demonstrated that SINEs, in addition to their established role as insulators [65], [66], may also influence gene expression in the brain. For example, AmnSINEs, a highly conserved family of SINEs identified in the genome of amniota, contribute to the expression of genes necessary for brain development, in some cases acting as tissue-specific enhancers [39], [40].

Although acetylated SINEs shared some properties with previously identified neuronal enhancers [16], including the presence of H3K4me1 and increased transcription in response to depolarization, they do not include the consensus sequences for the nuclear factors CREB, SRF or Npas4 (CP and AR, unpublished observations). Acetylated SINEs contained a motif that completely overlapped with the B box, the binding sequence for the general transcription factor TFIIIC [50]. Importantly, TFIIIC binding to acetylated SINEs located near the TSS of inducible genes regulated activity-dependent transcription. Silencing of the GTf3c5 subunit induced both SINE acetylation and unregulated transcription of inducible genes that resulted in a dramatic increase of dendritic growth and branching. Thus, the interaction of TFIIIC with acetylated SINEs may act as a “brake” of the transcriptional response, coordinating the expression of the many genes required for dendritic growth and neuronal differentiation.

**Figure 7:**
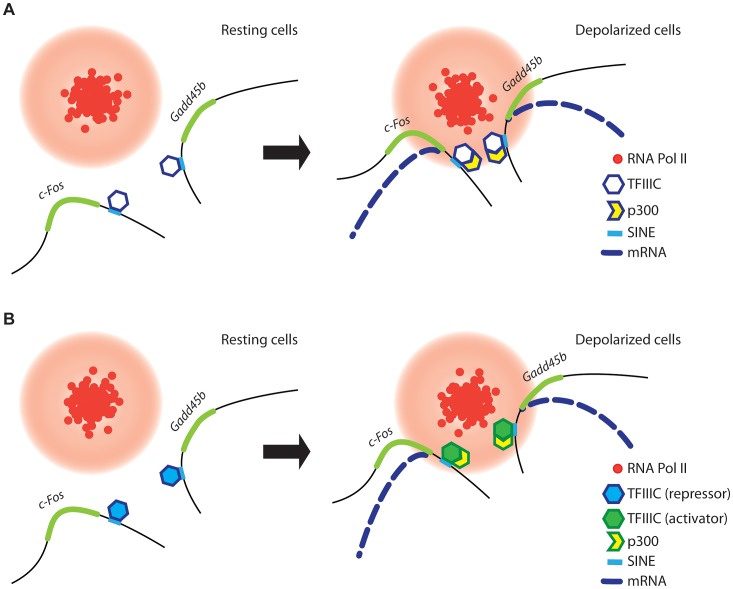
A model for TFIIIC-dependent regulation of activity-regulated transcription. (A and B) In resting conditions (left), TFIIIC is bound to SINEs located in the proximity of poised, activity dependent genes. TFIIIC keeps gene loci away from transcription factories, thereby inhibiting transcription. (A) In response to depolarization, p300 is recruited to SINEs and co-regulated genes come in close proximity within shared TFs, where they become transcriptionally activated. (B) Alternatively, upon depolarization TFIIIC undergoes a functional switch, p300 is recruited to SINEs and genes relocate to TFs where they become transcriptionally activated.

How does TFIIIC control gene relocation and transcription in neurons? In mammalian cells, genes that are activated either in response to stimulation or during cell differentiation preferentially relocate to shared TFs [22], [55]. The simultaneous relocation of activated genes to preassembled TFs enriched with RNAPII and other specific nuclear factors [22] ensures accurate and efficient transcription [19]. We found that the interaction of TFIIIC with SINEs located near activity-dependent neuronal genes controlled their relocation to TFs, where they were rapidly transcribed. The enhanced binding of Gtf3c1 and p300 at SINEs observed in response to depolarization (Figure 2F,G) may reflect the simultaneous relocation and clustering of several SINE and promoter elements at shared TFs, where the local concentration of transcription factors and co-activators increases significantly (Figure 7A).

Two subunits of the TFIIIC complex possess acetyltransferase activity [47] and we and others have found that the histone acetyltransferase p300 is recruited to TFIIIC binding sites (Figure 2G and [48]). Therefore, TFIIIC may mediate *de novo* acetylation of SINEs either directly or indirectly via interaction with p300. Although in non-stimulated neurons TFIIIC functions as a transcriptional “brake”, a subunit switch may occur within the complex in response to neuronal activity, which allows TFIIIC to become a platform for the recruitment of transcriptional co-activators (Figure 7B). As suggested by the silencing experiments, the dissociation of the Gtf3c5 subunit from the complex may induce such a functional switch. An additional mechanism may rely on the ability of TFIIIC to mediate the loading of condensins on DNA [67]. In all eukaryotes, condensins are tightly associated with chromatin and regulate the assembly and the maintenance of chromosomal structure during mitosis [68], [69]. Interaction of condensins with TFIIIC bound to SINEs, located near activity-dependent neuronal genes, may mediate rapid changes of nuclear structure that results in the release of gene loci from the nuclear periphery, where they are maintained in a repressed state, to TFs, where they are transcribed. Thus, our data provide a link between TFIIIC function and transcriptional activation in response to depolarization, and identify a new role for the hundreds of ETC sites scattered across the mammalian genome [25], [27].

Finally, in the nervous system, nuclear architecture undergo substantial modifications during the different stages of differentiation, presumably in response to developmental cues [70]. Interestingly, three-dimensional image reconstruction analyses demonstrated that nuclei of hippocampal neurons undergo dramatic infolding and possibly substantial chromatin re-organization following short-term burst of synaptic activation [71] and in humans, the X chromosome shows a dramatic nuclear repositioning in response to epileptic seizures [72]. One of the implications of our study is that in neurons, changes of nuclear architecture are required not only for long-lasting expression of specific genes in differentiated cells, but also for more rapid transcriptional responses.

## Materials and Methods

### Exposure to Novel Enriched Enviroment (NEE)

C57BL/6J female mice were separated after weaning and housed singly in conventional shoebox polycarbonate cages with *ad libitum* access to food pellets and water. Female mice (2 to 4 months of age) were used in order to avoid any aggressive behaviour caused by mating or territorial needs. Prior to the experiments, mice were gently handled for a few minutes every day for at least a week to minimize the stress associated with handling and to ensure that the mice were exclusively stimulated by exposure to enriched environmental conditions. On the day of the experiment, mice were randomly sorted into control (e.g. unstimulated) or stimulated groups, and stimulation was performed always in the late afternoon. Stimulation was achieved by moving the mice to a complex environment represented by two large, semi-transparent plastic boxes (80×40×50 cm each) joined by three pipes. The boxes were fitted with cardboard tubes, mazes and several structures to provide spatial complexity. Several items (including wood chips and paper towels) and food (seeds, dried and fresh fruit) were used for sensory stimulation. Social interactions were obtained by stimulating at least five mice simultaneously. While in the novel environment, mice displayed a consistent exploratory behaviour, occasionally feeding and drinking, and were constantly monitored for signs of stress (e.g. fighting, stereotypical behaviours). Mice were exposed to NEE for the indicated times, culled by cervical dislocation and brain dissection was performed immediately after culling. Dissected cortices were immediately frozen into dry ice-cooled isopentane and stored at −80°C. Control animals were culled on the day of the stimulation.

### Cell culture and transfection

Cortices of E15 mouse embryos were dissected in ice-cold HBSS, incubated with 20 U/ml Papain (Worthington) for 25 minutes at 37°C. Tissue was resuspended in adhesion medium (MEM supplemented with 10% FBS and 5% horse serum) and gently dissociated with a 5 ml serological pipette. Neurons were plated either on glass coverslips in 4 well-plates or on 10 cm dishes, and after 4 hours, culture medium was replaced with Neurobasal medium supplemented with B27. For Immuno-DNA FISH and RNA FISH experiments, neurons cultured on glass coverslips for 2 days were transfected using Optimem containing 200 ng of plasmidic DNA, 100 pmol of siRNA and 0.8 µl Lipofectamine2000 (Invitrogen). Either control siRNA (QIAGEN Allstar negative control, #1027281) or a pool of Gtf3c5 siRNA molecules (ON-TARGETplus SMARTpool L-056126-01-0005, Dharmacon; target sequences: gag gaa agc guc ucu cga a, gca cca auc cca uag auc a, acu gau ggc cca cgg aaa u, aca gag ugc uca ugc gca a) were used, in combination with an eGFP-actin or GFP expression vector. After 3 hours, transfection medium was replaced with culture medium and neurons were cultured for 72 hours before stimulation. Cells were either stimulated with 50 mM KCl for 45 minutes or left untreated. Prior to stimulation, cells were cultured overnight in low serum (30% compared to standard medium) in the presence of 50 µM AP5. Unless otherwise stated, Nifedipine (MP Biomedicals), TTX (Tocris Bioscience) and DRB (Sigma) were added to the culture medium 15 minutes prior to stimulation. Bicuculline was purchased from Tocris Bioscience. NIH/3T3 fibroblasts were cultured according to to ATCC guidelines and transfected using Lipofectamine2000 (Invitrogen) as per manufacturer's instructions. Cell culture reagents were purchased from Gibco.

### shRNA and expression vectors

shRNA sequences targeting either firefly Luciferase (shLuc, gct gac gcg gaa tac ttc gtt caa gag acg aag tat tcc gcg tca gc) or Gtf3c5 (shGtf3c5, gag gaa agc gtc tct cga att caa gag att cga gag acg ctt tcc tc) were cloned into pSUPER.neo+gfp vector (BglII/XhoI). To obtain a myc-tagged isoform, mouse Gtf3c5 coding sequence was cloned from pCMV-SPORT6-Gtf3c5 (Image clone IRAVp968E0646D, Source Bioscience) into pCMV-myc by blunt-end cloning and subsequently the siRNA-target region was mutagenized (referred to as myc-Gtf3c5R; Quickchange II site-directed mutagenesis, Stratagene). GFP coding sequence in pSUPER constructs was either excised (pSUPER-shLuc and pSUPER-shGtf3c5 constructs) or substituted with myc-Gtf3c5R coding sequence (Nhe/NotI), allowing the simultaneous silencing of endogenous Gtf3c5 and the expression of shRNA-resistant myc-Gtf3c5R.

### Lentivirus production and infection

shLuc and shGtf3c5 sequences were cloned into the GFP-containing lentiviral vector L303 (XhoI/XbaI; gift of A. Citri). Self-inactivating HIV lentivirus particles were produced by transfecting HEK293T cells with the vector, envelope and packaging plasmids (pCMV_VSV_G and Δ8.9, Addgene). Mouse primary cortical neurons were infected 6 hours after plating, and medium was changed after 16 hours. 5 days after infection, neurons were either stimulated with 50 mM KCl for 45 minutes or left untreated, and subjected to RNA extraction, cDNA synthesis and expression analyses.

### Chromatin immunoprecipitation

Somatosensory cortices dissected form either control or NEE-exposed mice were fixed in 1% formaldehyde in PBS for 15 minutes. Cross-linking reaction was terminated by adding glycine to a final concentration of 125 mM. After homogenisation, dissociated cells were harvested by centrifugation and resuspendend in sonication buffer (20 mM Tris-HCl pH 8.1, 150 mM NaCl, 0.1% SDS, 0.5% Triton X-100). Alternatively, dissociated cells were resuspended in cell lysis buffer (5 mM PIPES pH 8.0, 85 mM KCl, 0.5% NP40) and incubated up to 15 minutes on ice. Nuclei were purified by centrifugation (5 minutes at 1000×g), lysed in nuclei lysis buffer (50 mM Tris-HCl pH 8.1, 10 mM EDTA, 1% SDS), and incubated 20 minutes on ice. Lysates were frozen in liquid N_2_ and thawed twice prior to sonication. For *in vitro* experiments, at least 5×10∧6 primary cortical neurons were fixed in 1% formaldehyde in PBS for 20 minutes, followed by inactivation with 125 mM glycine. Cells were harvested in 100 mM Tris-HCl pH 9.4, 10 mM DTT, centrifuged and resuspended in sonication buffer. Sonicaton was performed using a Bioruptor (Diagenode), by applying 30 pulses, 30 seconds each, at 30 seconds intervals, thus shearing chromatin into fragments 200–400 nt in size. After centrifugation at 13,000 g for 10 minutes at 4°C, supernatants were pre-cleared with Protein A-Sepharose beads (GE Healthcare, 1 hour at 4°C) prior to immunoprecipitation with anti-Histone H3K9K14ac (06-599, Millipore), anti-Histone H3 (ab10799, Abcam), anti-Gtf3c1 (A301-291A, Bethyl Laboratories), anti-p300 (Santa Cruz, sc-585X) or control IgG (Santa Cruz), overnight at 4°C. Immune complexes were purified by incubating the lysates with Protein A-Sepharose beads for 1 hour at 4°C, followed by 5 washes in 50 mM Hepes pH 7.6, 1 mM EDTA, 0.5 M LiCl, 0.7% sodium deoxycholate, 1% NP40, and 2 washes in 10 mM Tris-Hcl ph 8.1, 1 mM EDTA, 5 minutes each. All buffers contained protease and phosphatase 2 and 3 inhibitor cocktails (Sigma). Beads were boiled with 10% Chelex 100 resin (Bio-Rad) and DNA was eluted with 10 mM Tris-HCl pH 8.0. Alternatively, DNA was eluted by washing the beads twice for 15 min in 0.1 M NaHCO_3_ (pH 8.0), 1% SDS, followed by 16 hours incubation at 65°C in the presence of 300 mM NaCl and subsequent purification by PCR purification columns (Qiagen), according to manufacturer's instructions. Total input DNA was purified by boiling for 15 minutes an aliquot of sonicated chromatin in the presence of 300 mM NaCl, followed by phenol-chlorophorm extraction. DNA was subjected to quantitative real-time PCR in 25 µl reactions containing 12.5 µl of DyNAmo Flash SYBR Green qPCR Kit (Thermo Scientific) and 0.2 µM primers. All reactions were performed in triplicate with an Eppendorf Mastercycler Realplex 2 and each experiment included a standard curve and a no-template control. Standard curves consisted of serial dilutions of gel-purified PCR amplicons of known concentration. At the end of 40 cycles of amplification, a dissociation curve was obtained in which SYBR Green fluorescence was measured at 1°C intervals between 60°C and 100°C. Melting temperatures of amplicons varied between 79 and 83°C. For Gtf3c1 ChIP experiments (Figure 2D,E), negative control primers were designed on a 2 kbp region of chromosome 12 devoid of any H3K9K14ac and SINE. Primer sequences are listed in Table S4.

### Chromatin immunoprecipitation and sequential analysis of gene expression (ChIPseq)

Chromatin was purified from the somatosensory cortex of mice either unstimulated or exposed to NEE for 45 minutes. Steps were taken to reduce potential sources of variability due to individual animals and antibody efficiency. Four (control) or six (NEE) age-matched mice were used for each experimental condition. Rather than performing a single immunoprecipitation for each sample, aliquots of chromatin were pooled into 4 separate immunoprecipitation reactions taking care to use the same amount of chromatin from each cortex. The genomic DNA obtained from the 4 immunoprecitations was pooled and subjected to high-throughput sequencing. For each condition, at least 7 millions tags were sequenced using the Illumina GA-II platform at Fasteris SA (Switzerland). Reads were mapped to the mouse genome (release NCBIm37) using MAQ 0.6.6 (http://maq.sf.net/). Reads with potentially ambiguous mappings were discarded (MAQ quality score threshold > = 10). To avoid bias due to potential clonality artifacts, we also discarded any second and subsequent mappings to a given position on the genome (i.e. all mappings used in subsequent analyses must be derived from independent molecules). To determine regions of differential acetylation (referred here as +Δac and −Δac) all reads mapping within 2 kb sliding windows across the genome were counted and differential enrichment relative to a Binomial null model was analyzed (parameters used: p = total_size_of_library_1/[total_size_of_library_1 + total_size_of_library_2]). This approach excluded the detection of artifacts deriving from the presence of non-specific immunoprecipitated DNA, thus making a negative control ChIP unnecessary. The number of reads (Table S1) was sufficient to provide statistical robustness, as the false discovery rate estimated from the binomial model employed to identify the regions of differential acetylation was 0.040. Windows with a score of p<10∧-3 were considered for subsequent analysis.

### RNA extraction and gene expression analysis

Total RNA from somatosensory cortices of mice or from cultured primary cortical neurons were extracted using Trizol (Invitrogen), according to the company specifications. Genomic DNA was removed by treating the samples with DNAse I (Roche) for 30 minutes, followed by phenol/chloroform purification. For expression analysis by quantitative real-time PCR, 1–2 µg of total RNA were reversed-transcribed by using Superscript III (Invitrogen) and analysed by using DyNAmo Flash SYBR Green qPCR Kit (Thermo Scientific), as performed for ChiP-qPCR experiments (see above). Primer sequences are listed in Table S4. For the microarray experiments, age-matched female mice were separated into control and NEE-stimulated groups, and 3 mice were used for each experimental condition. Expression analysis was performed by Cambridge Genomic Services (Cambridge, UK). Quality of total RNA was verified using Agilent 2100 Bioanalyzer followed by expression analysis using a MouseWG-6 Expression BeadChip (Illumina).

### Motif inference and analysis

To identify DNA motifs over-represented in +Δac regions the motif prediction tool NestedMICA was employed [35]. A background model was generated using randomly selected genomic sequences of size comparable to +Δac regions. For sets of +Δac regions overlapping TSSs, the background sequences were also encompassing TSSs. The pool of analysed regions was randomly split into “test” and “validation” sets. Motif prediction was performed on the test set. To exclude false positives, over-representation of the obtained motifs was verified in the validation set. Predicted motifs showing over-representation in both test and validation sets, as compared to the background, were considered for further analysis.

### Immuno-DNA Fluorescence *In Situ* Hybridization (FISH)

Immuno-DNA FISH experiments were performed as previously described [73] with modifications. Briefly, cells were fixed for 30 minutes in PBS containing 0.5% glutaraldehyde, 0.3% Triton-X 100, followed by two 15 minutes treatments with 7.5% NaBH_4_. After blocking with 5% normal goat serum and 5% fetal bovine serum, RNAPII-ser5P (4H8, 05-623 Millipore) and GFP (ab6556, Abcam) antibodies were applied, followed by detection with secondary antibodies detection (Anti-mouse Alexa568, anti-rabbit Alexa488, Molecular Probes), post-fixation with 50 mM EGS for 30 minutes at 37°C and 100 mg/ml RNAse A treatment for 1 hour. Between each step, coverslips were washed extensively with PBS. Nuclear genomic DNA denaturation was obtained by incubating the coverslips in 70% formamide in 0.2× SSC for 30 minutes at 80°C, followed by hybridization with a digoxigenin-labeled probe for 16 hours at 38°C. Probes were labelled with digoxigenin-dUTP using a nick translation kit (both from Roche). 0.1 µg DNA probe was pre-annealed with mouse Cot-1 DNA for 45 minutes at 37°C and denatured 10 minutes at 95°C immediately before hybridization. The following BAC clones (CHORI) were used to generate digoxigenin-labeled probes: *c-Fos*, RP24-233K8; *Gadd45b*, RP23-382P20; *Csn2*, RP23-110B6; *Gapdh*, RP23-268O22; *Bdnf*, RP24-149F11; *Fcf1*, RP24-185C13. Confocal images of neuronal nuclei were acquired using a Leica TCS SP5 confocal microscope (z-distance 0.2 µm). Images were analysed using Fiji and an appositely developed algorithm. No blinding was required as the analysis was performed computationally, with limited pre-processing of the images. The 3D Objects Counter tool was used to identify each DNA FISH signal and measure the coordinates of its centre of mass, based both on the signal shape and the intensity of each voxel. The 3D Objects Counter tool was employed also to identify transcription factories. RNAPII-ser5P foci were identified by applying a threshold based on mean and standard deviation of the voxels within the cell nucleus (counterstained by DAPI), to account for the small variability of fluorescence intensity between different cells and experiments. Based on the threshold level (calculated as mean +1 S.D.) voxels were defined either as RNAPII “positive” or “negative”. Gene locus-to-TF distance was determined by using an algorithm that calculated the distance between the centre of mass of the DNA FISH signal and the nearest of the “RNAPII positive” voxels, in all directions and not only on the same confocal plane. The co-localization threshold was set to 225 nm, corresponding to the distance at which the two smallest detectable objects overlap. Co-localization of FISH signals and TFs was verified by visual inspection after the analysis.

### RNA Fluorescence *In Situ* Hybridization (FISH)

Mouse primary cortical neurons were fixed for 30 minutes with 4% PFA in PBS, followed by a 5 minutes wash with 0.1% DEPC in PBS and a 20 minutes permeabilization step with 0.3% Triton X-100 in PBS. After each step, coverslips were washed extensively with PBS. All solutions were RNAse-free. After pre-hybridization for 2 hours at room temperature in hybridization solution (Sigma), coverslips were incubated for 16 hours at 55°C with hybridization solution containing 500 ng/ml of digoxigenin-labeled cRNA probe. Probes were labelled with digoxigenin-UTP by performing *in vitro* transcription (Roche) of a linearized plasmidic DNA template encoding *c-Fos* coding sequence (NM_010234). Prior to hybridization, probes were denatured for 5 minutes at 85°C. Excess probe was washed using increasingly stringent conditions (5 minutes at 55°C in 5× SSC, 1 minute at 55°C in 2× SSC, 30 minutes at 55°C in 0.2× SSC with 50% formamide, 5 minutes at room temperature in 0.2× SSC) and coverslips were incubated with 1% Blocking reagent (Roche) in 250 mM NaCl, 100 mM Tris-HCl pH 7.5 for 1 hour, followed by consecutive incubation with primary (anti-digoxigenin alkaline phosphatase conjugated from sheep, Roche; anti-GFP from rabbit, Abcam) and secondary antibodies (anti-rabbit Alexa488, Invitrogen). Anti-digoxigenin immunoreactivity was detected by applying the alkaline phosphatase substrate Fast Red (Roche), according to the manufacturer's instruction. Confocal images of neuronal soma were acquired using a Leica TCS SPE confocal microscope and analysed using Fiji. 3D Objects Counter tool was employed to identify c-Fos ribonucleoparticles and measure their fluorescence intensity. Ribonucleoparticles located within transfected cells only were identified using the GFP counterstaining.

### Dendritogenesis assay

3 hours after plating, mouse cortical neurons were transfected using Optimem containing 400 ng of GFP expression vector (no siRNA control), or 200 ng GFP vector and 100 pmol of siRNA (control and Gtf3c5 siRNA) and 0.8 µl Lipofectamine2000 (Invitrogen). After 3 hours, medium was replaced with culture medium with or without 50 mM KCl. Cells were cultured for 2 days followed by immunostaining with anti-GFP (ab6556, Abcam) and anti-MAP2 (M9942, Sigma) antibodies. Dendrites were identified by MAP2 staining. Images were obtained using a Zeiss Axioplan 2 microscope and analysed in Fiji. For Sholl analysis and quantification of total dendritic length we used the Simple Neurite Tracer plugin. Images of the dendritic profiles shown in Figure 6A were generated using the Trainable Segmentation plugin (original images are provided in Figure S6A).

### *In Situ* hybridization

Brains of control and NEE-exposed mice were frozen in dry ice-cold isopentane and stored at −80°C until cryosectioning. 12 µm coronal sections were cut using a Leica CM1850 cryostat. Sections were fixed in PBS containing 4% paraformaldehyde for 20 minutes, followed by 10 minutes incubation with 1% triethanolamine, 0.25% acetic anhydride and 2 h prehybridization with hybridization solution (Sigma). Between each step, slides were washed extensively in PBS. Samples were incubated overnight at 55°C with 500 ng/ml of digoxigenin-labeled probe in hybridization solution and washed as for fluorescent in situ hybridization (see above). Slides were incubated with 1% Blocking reagent (Roche) for 1 hour, followed by anti-digoxigenin-POD conjugate antibody and NBT/BCIP colorimetric reaction.

### Immunofluorescence

Cortical neurons were cultured on glass coverslips, fixed with 4% PFA in PBS for 20 minutes, permeabilized with 0.3% Triton X-100 in PBS and subjected to immunostaining using anti Gtf3c5 (A301-242A, Bethyl Laboratories), anti-GFP (ab13970, Abcam) and anti-c-Myc (ab32, Abcam). Cell nuclei were identified using DAPI.

### Western blotting

Cells were harvested, resuspended in RIPA buffer (50 mM Tris pH 7.5, 150 mM NaCl, 1 mM EDTA, 1% NP40, 0.5% sodium deoxycholate, 0.1% SDS), incubated 20 minutes on ice and centrifuged 20 minutes at 13,000×g. Mouse cortex samples were homogenized in RIPA buffer and processed as above. Supernatants were denatured in Laemmli buffer followed by SDS-PAGE and western blotting analysis. Antibodies used were Gtf3c1 (NB100-60657, Novus Biologicals), Gtf3c2 (A301-236A, Bethyl Laboratories), Gtf3c3 (A301-238A, Bethyl Laboratories), Gtf3c4 (A301-239A, Bethyl Laboratories), Gtf3c5 (A301-242A, Bethyl Laboratories), Gtf3c6 (ab107804, Abcam), c-Myc (sc56634, Santa Cruz), Hsp90 (sc1055, Santa Cruz), Gapdh (ab9494, Abcam).

### Ethics statement

All animal work must have been conducted according to relevant legislation in United Kingdom (Animals Scientific Procedures Act 1986).

## Supporting Information

Dataset S1 Profile of acetylation changes in response to NEE. The wig file H3K9K14ac_changes.wig can be uploaded to the UCSC Mouse Genome Browser (genome.ucsc.edu). Regions of significant H3K9K14ac increase are shown as black column bars. The height of each column is −log(p-value). Regions of significant H3K9K14ac decrease are shown as grey column bars. The height of each column is log(p-value). Only regions with p-value<0.001 are included (provided as a compressed WIG file).
(ZIP)

Figure S1 (A) Images of the environmental enrichment experimental settings. (B) *In situ* hybridisation assay performed on coronal sections of somatosensory cortex and hippocampus of adult mice, either exposed to NEE conditions for the indicated time or left untreated. (C) qRT-PCR analyses of genes induced by NEE. Adult mice were either exposed to NEE for 3 hours or left untreated, somatosensory cortex was dissected and subjected to total RNA extraction and cDNA synthesis, followed by qRT-PCR. Shown are the expression levels of Arc, c-Fos and Gadd45b normalized to the housekeeping gene Gapdh (at least 4 mice were used for each experimental condition; *, P<0.05, ***, P<0.001, Student's t-test). (D) Genomic context of NEE-dependent acetylation changes. Regions of increased (left column) or decreased (right column) H3K9K14ac were classified either as overlapping with a transcription start sites (TSS), with a gene body or extragenic. (E) Venn diagram of the overlap between genes with increased levels of H3K9K14ac at TSS, with genes whose mRNA was increased by NEE exposure (NI), based on microarray expression profiling. (F) Ac1 (blue) and Ac2 (red, green) motifs aligned to the consensus sequence of mouse B1 SINEs (taken from the database of repetitive DNA elements Repbase Update, http://www.girinst.org/repbase/). A and B boxes are indicated. (G) Histogram representing the frequency of elements of SINE classes, individual SINE families and LINE classes in +Δac regions, when compared to a randomly selected set of genomic regions of comparable size (bg). Frequency is expressed as number of elements every 1000 regions (*, P<0.05; ***, P<0.001; Fisher's exact test). (H) Sequences of *c-Fos* ^RSINE1^ and *Gadd45b* ^B1F^. The SINEs located downstream of *c-Fos* and *Gadd45b* harbour a putative B box (boldface, underlined).
(TIF)

Figure S2 Histone H3 acetylation at *c-Fos* ^RSINE1^ and *Gadd45b* ^B1F^ increased in response to depolarization. Mouse primary cortical neurons were either exposed to 50 mM KCl for 45 min or left untreated, and subjected to ChIP using either H3K9K14ac or histone H3 antibodies, followed by qPCR. Histograms show the ratio of immunoprecipitation efficiency between H3K9K14ac and H3 antibodies relative to total chromatin input (average and s.e.m. of 4 experiments are shown; *, P<0.05, Student's t-test).
(TIF)

Figure S3 (A) Mouse primary cortical neurons were either exposed to 50 mM KCl for 45 minutes or left untreated, and subjected to RNA fluorescent in situ hybridisation. c-Fos mRNA particles are shown in red and cell nuclei stained with DAPI are in blue. Shown are maximal z-projections of confocal scans of samples hybridised with antisense c-Fos coding sequence probe and, as negative controls, with c-Fos sense probe or treated with RNAse prior hybridisation. (B) Consecutive confocal sections of primary neurons analysed by RNA-FISH with c-Fos antisense probe, shown in (A). mRNA particles (red) were detectable exclusively outside nuclei, as assessed by DAPI counterstaining (blue). Scale bars 5 µm. (C) The enhancement of c-Fos expression induced by silencing of Gtf3c5 is not depolarization-dependent. Two days after plating, mouse primary cortical neurons were transfected with control or Gtf3c5 siRNA in combination with a GFP expression vector, and 5 µM nifedipine was added. After three days, neurons were stimulated with 50 mM KCl for 45 minutes or left untreated and analysed by quantitative RNA-FISH. Culture in presence of nifedipine did not prevent the increase in c-Fos expression, induced by Gtf3c5 silencing. Histograms show the average and s.e.m. of the fluorescence intensity of at least 25 cells per condition (*, P<0.05, two-way ANOVA). (D) Mouse primary cortical neurons were infected 6 hours after plating with lentiviral particles encoding short hairpin RNAs that targeted either firefly luciferase (shLUC, negative control) or Gtf3c5 (shGtf3c5), and GFP to assess the efficiency of infection. Approximately 60% of the neurons showed GFP expression, as verified by immunostaining (LC, WTS and AR, unpublished observations). Four days after infection, cells were subjected to RNA extraction and cDNA synthesis followed by qRT-PCR analysis of Gtf3c5 mRNA and 5s rRNA. Gtf3c5 transcript was reduced to 43.8±5.2%, when compared to control, whereas 5s RNA expression showed no significant change. (E, F) Western blot analysis of the expression of TFIIIC subunits in the cortex of mice exposed to NEE for 45 minutes (E) and in mouse primary cortical neurons exposed to 50 mM KCl for the time indicated (F). Both *in vivo* and *in vitro*, no significant change was detected for any of the six TFIIIC subunits. (G) KCl depolarization does not affect cell viability. DIV5 mouse primary cortical neurons were exposed to 50 mM KCl for 45 minutes or left untreated, followed by DAPI staining of nuclear DNA. Neurons presenting nuclear collapse and clumps of condensed chromatin were counted as necrotic (n = 250 nuclei from 5 separate experiments).
(TIF)

Figure S4 *c-Fos* relocation to TFs is depolarization-dependent. (A, B) Box and whiskers plot of the distribution of the distance between *c-Fos* locus and the nearest TF. Whiskers denote the 90th and 10th percentiles, box edges denote the 75th and 25th percentiles, solid lines denote medians, dashed lines denote averages. For each condition 30 to 41 FISH signals were analysed. (A) DIV10 cortical primary neurons were stimulated with 50 µM bicuculline for 45 minutes, either in the presence of 1 µM tetrodotoxin (TTX) or alone, and analysed by immuno-DNA FISH. (B) Cortical primary neurons were stimulated with 50 mM KCl for 45 minutes, either in the presence of 5 µM nifedipine, 50 µg/ml DRB or alone, and analysed by immuno-DNA FISH.
(TIF)

Figure S5 (A) Mouse cortical neurons were cultured for 2 days and transfected either with control or Gtf3c5 siRNA, in combination with an eGFP-actin expression vector. After three days, cells were fixed and subjected to Gtf3c5 and GFP immunofluorescence staining. Single confocal sections are shown (scale bar 5 µm). (B, C and D) Two days after plating, mouse primary cortical neurons were transfected with control or Gtf3c5 siRNA in combination with an eGFP-actin expression vector. After three days, neurons were stimulated with 50 mM KCl for 45 minutes or left untreated and analysed by immuno-DNA FISH targeting *c-Fos*, *Gadd45b* and *Csn2* loci. Box and whiskers plots show the distribution of the distance between *c-Fos* (B), *Gadd45b* (C) and *Csn2* (D) gene loci and the nearest TF. Whiskers denote the 90th and 10th percentiles, box edges denote the 75th and 25th percentiles, solid lines denote medians, dashed lines denote averages. For each condition 32 to 44 FISH signals were analysed. (E) *c-Fos* relocation to TFs induced by silencing of Gtf3c5 is not depolarization-dependent. Two days after plating, mouse primary cortical neurons were transfected with control or Gtf3c5 siRNA in combination with an eGFP-actin expression vector, and 5 µM nifedipine was added. After three days, neurons were stimulated with 50 mM KCl for 45 minutes or left untreated and analysed by immuno-DNA FISH targeting *c-Fos* locus. Culture in presence of nifedipine did not prevent the relocation of *c-Fos* to TFs, induced by Gtf3c5 silencing. Histograms show the percentage of co-localization with TFs of *c-Fos* gene locus (*, P<0.05, Fisher's exact test; n = 30 to 42 FISH signals per condition). (F) Western blot analysis of Gtf3c5 expression in NIH-3T3 cells transfected with pSUPER constructs expressing either short hairpin RNA targeting firefly Luciferase (shLuciferase, as a negative control), shRNA targeting mouse Gtf3c5 (shGtf3c5), shLuciferase and myc-tagged Gtf3c5 mutated to be shRNA resistant (myc-Gtf3c5R), or shGtf3c5 and myc-Gtf3c5R. Expression of shGtf3c5 efficiently silenced Gtf3c5 (lane 3). Ectopic expression of myc-Gtf3c5R, in combination with shGtf3c5, successfully rescued the silencing (lane 5). (G) Immunofluorescence of mouse cortical neurons transfected with pSUPER constructs expressing either shLuciferase (as a negative control), shGtf3c5, or shGtf3c5 and myc-Gtf3c5R, in combination with an eGFP-actin expression vector. Single confocal sections are shown (scale bar, 5 µm). Expression of shGtf3c5 efficiently silenced Gtf3c5, whereas ectopic expression of myc-Gtf3c5R successfully rescued the silencing. (H) The expression of a siRNA-resistant form of Gtf3c5 rescued the effect of Gtf3c5 silencing on the relocation of *c-Fos* to TFs. Mouse cortical neurons were transfected with pSUPER constructs expressing either shLuciferase, shGtf3c5, or shGtf3c5 and myc-Gtf3c5R, in combination with an eGFP-actin expression vector. After three days cells were exposed to KCl or left untreated, followed by immuno-DNA FISH analysis, targeting *c-Fos* locus and RNAPII-ser5P transcription factories. Histograms show the percentages of *c-Fos* co-localization with TFs (*, P<0.05, Fisher's exact test; n = 42 to 50 FISH signals per condition).
(TIF)

Figure S6 Sholl analysis of cortical neurons. (A) Original images of cortical neurons (shown in Figure 6A) transfected with a GFP-expressing vector alone or in combination with either control or Gtf3c5 siRNA. Neurons were cultured for 2 days in basal conditions or in presence of 50 mM KCl, followed by GFP immunostaining. Sholl profiles of neurons untransfected (B), transfected with a control siRNA (C) or with Gtf3c5 siRNA (D), and maintained for two days in basal conditions (dashed line) or exposed to KCl (solid line). For each distance point, the average number of intersections and s.e.m. are shown. At least 25 cells per condition were analysed (*, P<0.05; **, P<0.01; ***, P<0.001, two-way ANOVA).
(TIF)

Table S1 Summary of ChIPseq analysis.
(DOC)

Table S2 Transcripts regulated in the mouse somatosensory cortex following 3 hours of NEE stimulation, as compared to untimulated control. For each transcript, fold change, log2(fold change), p-value and adjusted p-value are shown. 196 up-regulated and 70 down-regulated transcripts with adjusted p-value<0.05 are listed.
(XLS)

Table S3 Genomic coordinates of SINEs analysed.
(DOC)

Table S4 Primer sequences.
(DOC)
