# Supplementary figures and images for: Binding of TFIIIC to SINE Elements Controls the Relocation of Activity-Dependent Neuronal Genes to Transcription Factories
Source: PLoS Genet. 2013 Aug 15;9(8):e1003699. doi: 10.1371/journal.pgen.1003699 (PMC3744447; doi:10.1371/journal.pgen.1003699)

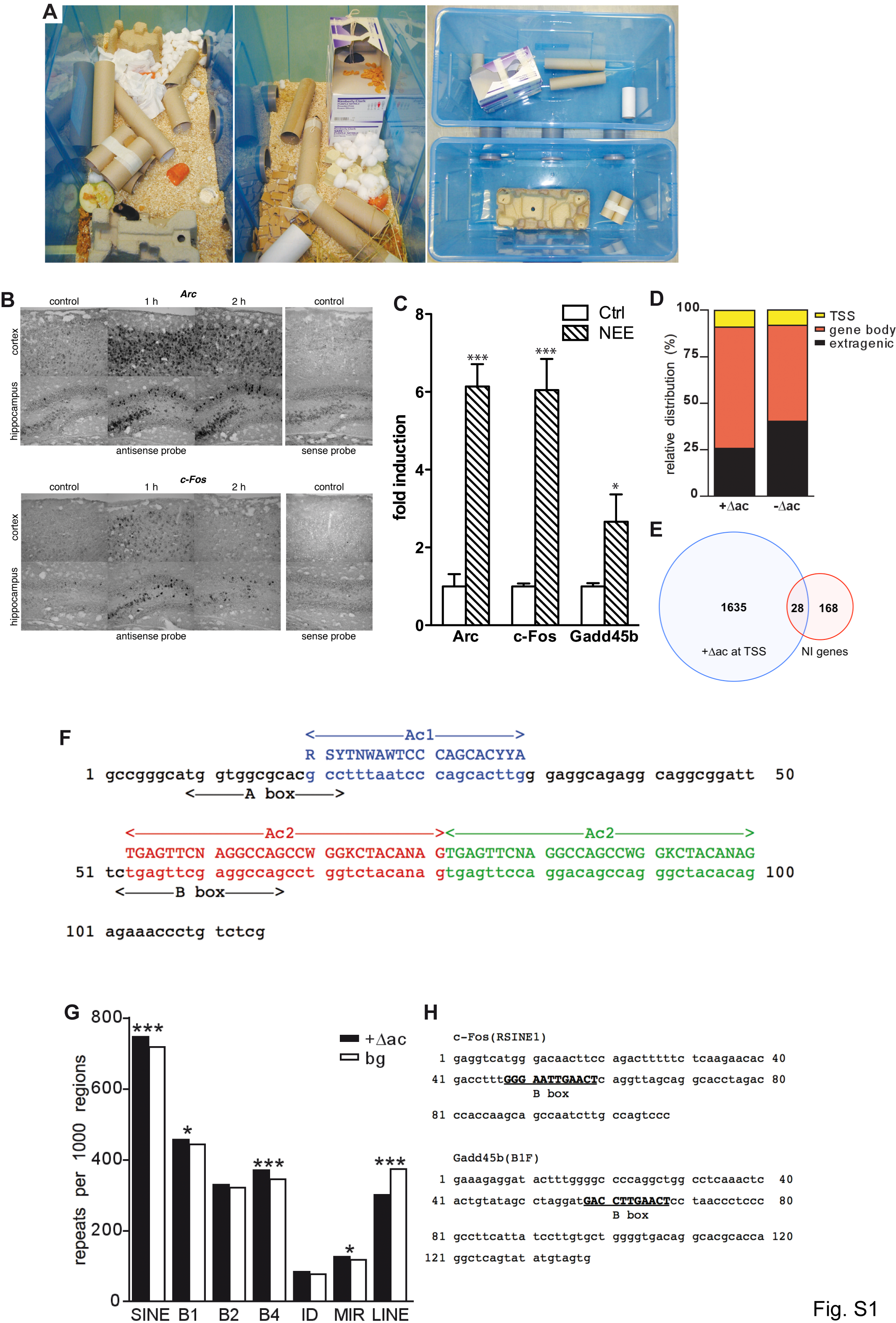

Supplement: Figure S1 — (A) Images of the environmental enrichment experimental settings. (B) In situ hybridisation assay performed on coronal sections of somatosensory cortex and hippocampus of adult mice, either exposed to NEE conditions for the indicated time or left untreated. (C) qRT-PCR analyses of genes induced by NEE. Adult mice were either exposed to NEE for 3 hours or left untreated, somatosensory cortex was dissected and subjected to total RNA extraction and cDNA synthesis, followed by qRT-PCR. Shown are the expression levels of Arc, c-Fos and Gadd45b normalized to the housekeeping gene Gapdh (at least 4 mice were used for each experimental condition; *, P<0.05, ***, P<0.001, Student's t-test). (D) Genomic context of NEE-dependent acetylation changes. Regions of increased (left column) or decreased (right column) H3K9K14ac were classified either as overlapping with a transcription start sites (TSS), with a gene body or extragenic. (E) Venn diagram of the overlap between genes with increased levels of H3K9K14ac at TSS, with genes whose mRNA was increased by NEE exposure (NI), based on microarray expression profiling. (F) Ac1 (blue) and Ac2 (red, green) motifs aligned to the consensus sequence of mouse B1 SINEs (taken from the database of repetitive DNA elements Repbase Update, http://www.girinst.org/repbase/). A and B boxes are indicated. (G) Histogram representing the frequency of elements of SINE classes, individual SINE families and LINE classes in +Δac regions, when compared to a randomly selected set of genomic regions of comparable size (bg). Frequency is expressed as number of elements every 1000 regions (*, P<0.05; ***, P<0.001; Fisher's exact test). (H) Sequences of c-Fos RSINE1 and Gadd45b B1F. The SINEs located downstream of c-Fos and Gadd45b harbour a putative B box (boldface, underlined). (TIF) [file pgen.1003699.s002.tif]

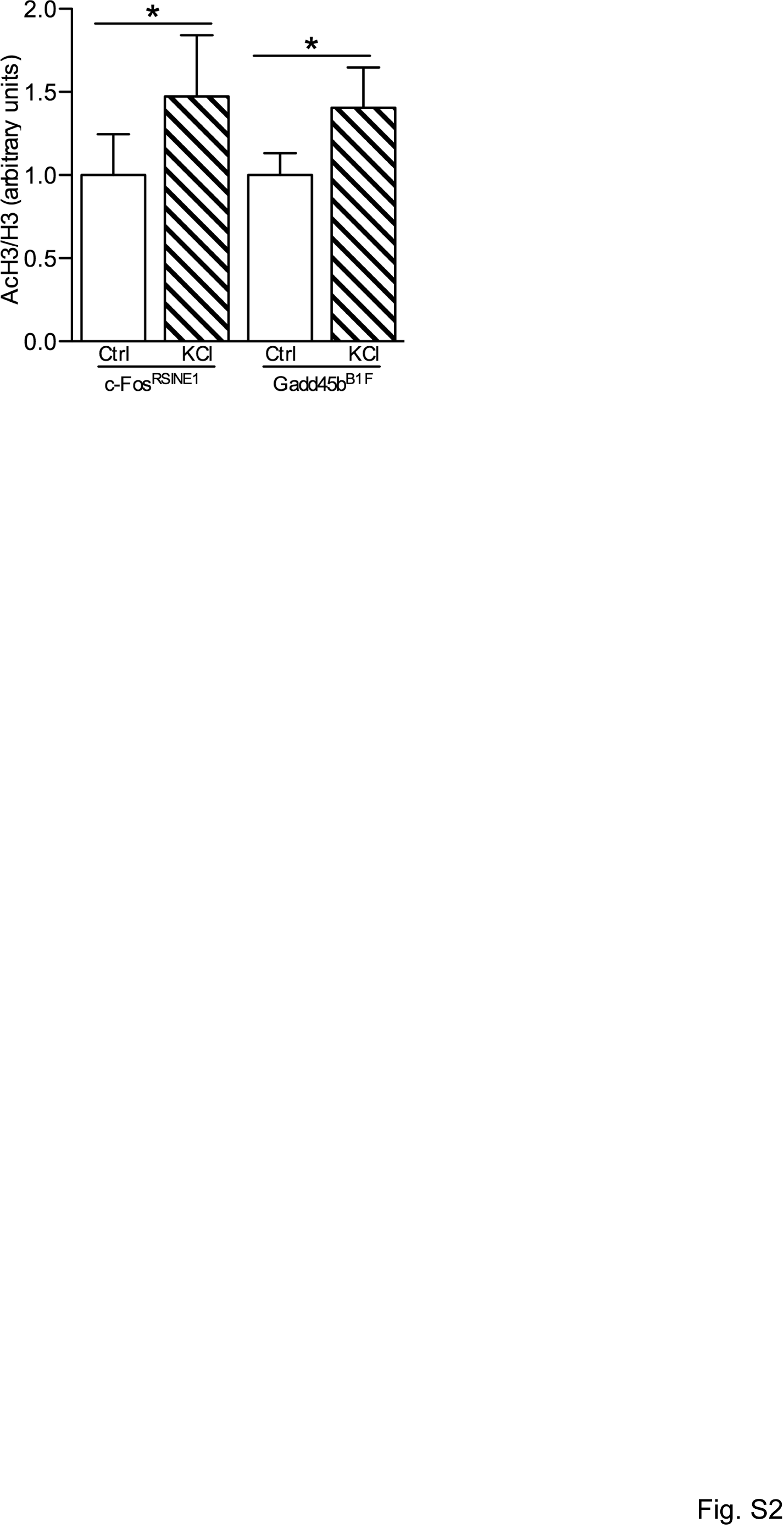

Supplement: Figure S2 — Histone H3 acetylation at c-Fos RSINE1 and Gadd45b B1F increased in response to depolarization. Mouse primary cortical neurons were either exposed to 50 mM KCl for 45 min or left untreated, and subjected to ChIP using either H3K9K14ac or histone H3 antibodies, followed by qPCR. Histograms show the ratio of immunoprecipitation efficiency between H3K9K14ac and H3 antibodies relative to total chromatin input (average and s.e.m. of 4 experiments are shown; *, P<0.05, Student's t-test). (TIF) [file pgen.1003699.s003.tif]

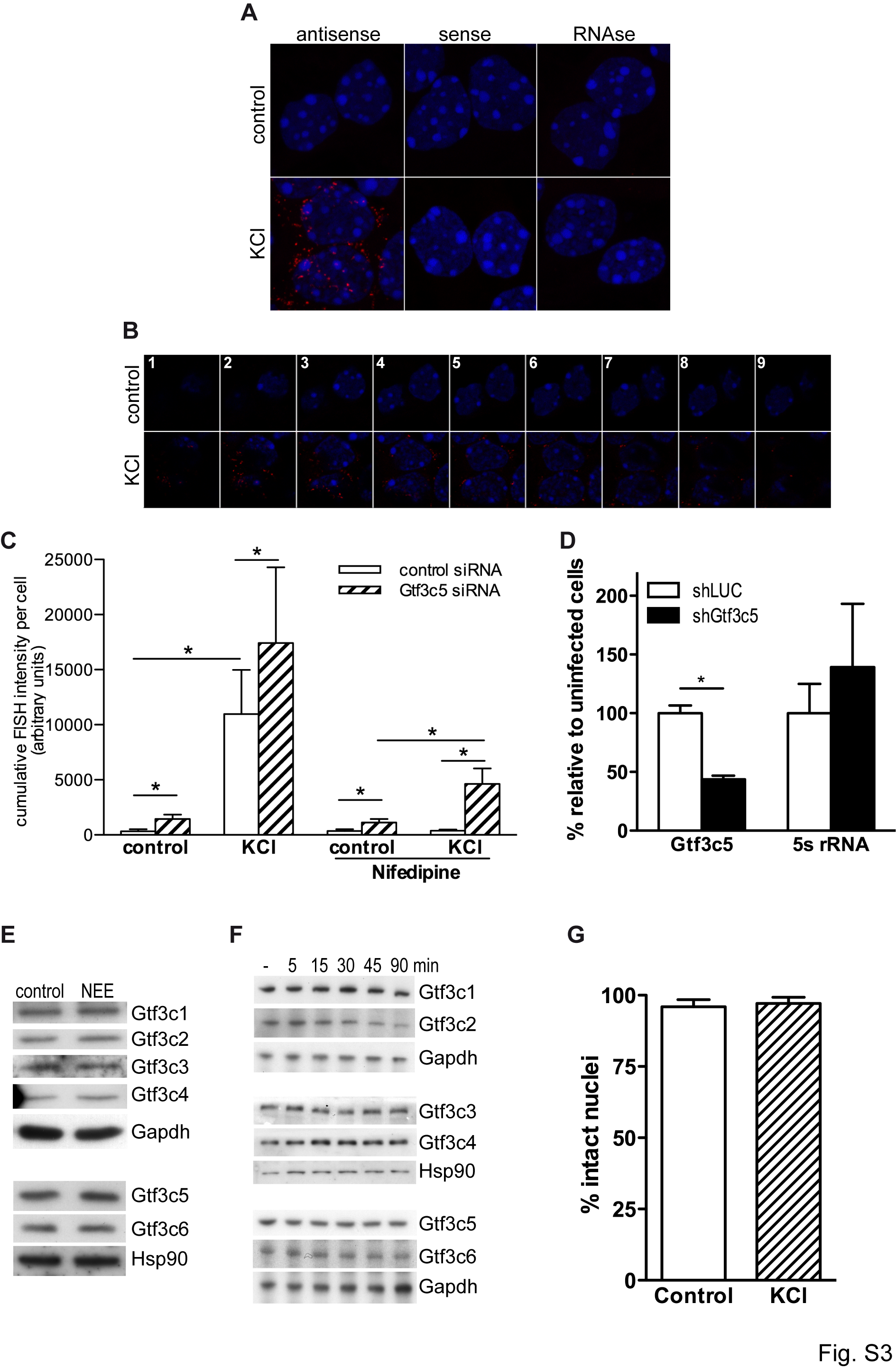

Supplement: Figure S3 — (A) Mouse primary cortical neurons were either exposed to 50 mM KCl for 45 minutes or left untreated, and subjected to RNA fluorescent in situ hybridisation. c-Fos mRNA particles are shown in red and cell nuclei stained with DAPI are in blue. Shown are maximal z-projections of confocal scans of samples hybridised with antisense c-Fos coding sequence probe and, as negative controls, with c-Fos sense probe or treated with RNAse prior hybridisation. (B) Consecutive confocal sections of primary neurons analysed by RNA-FISH with c-Fos antisense probe, shown in (A). mRNA particles (red) were detectable exclusively outside nuclei, as assessed by DAPI counterstaining (blue). Scale bars 5 µm. (C) The enhancement of c-Fos expression induced by silencing of Gtf3c5 is not depolarization-dependent. Two days after plating, mouse primary cortical neurons were transfected with control or Gtf3c5 siRNA in combination with a GFP expression vector, and 5 µM nifedipine was added. After three days, neurons were stimulated with 50 mM KCl for 45 minutes or left untreated and analysed by quantitative RNA-FISH. Culture in presence of nifedipine did not prevent the increase in c-Fos expression, induced by Gtf3c5 silencing. Histograms show the average and s.e.m. of the fluorescence intensity of at least 25 cells per condition (*, P<0.05, two-way ANOVA). (D) Mouse primary cortical neurons were infected 6 hours after plating with lentiviral particles encoding short hairpin RNAs that targeted either firefly luciferase (shLUC, negative control) or Gtf3c5 (shGtf3c5), and GFP to assess the efficiency of infection. Approximately 60% of the neurons showed GFP expression, as verified by immunostaining (LC, WTS and AR, unpublished observations). Four days after infection, cells were subjected to RNA extraction and cDNA synthesis followed by qRT-PCR analysis of Gtf3c5 mRNA and 5s rRNA. Gtf3c5 transcript was reduced to 43.8±5.2%, when compared to control, whereas 5s RNA expression showed no significant ch [file pgen.1003699.s004.tif]

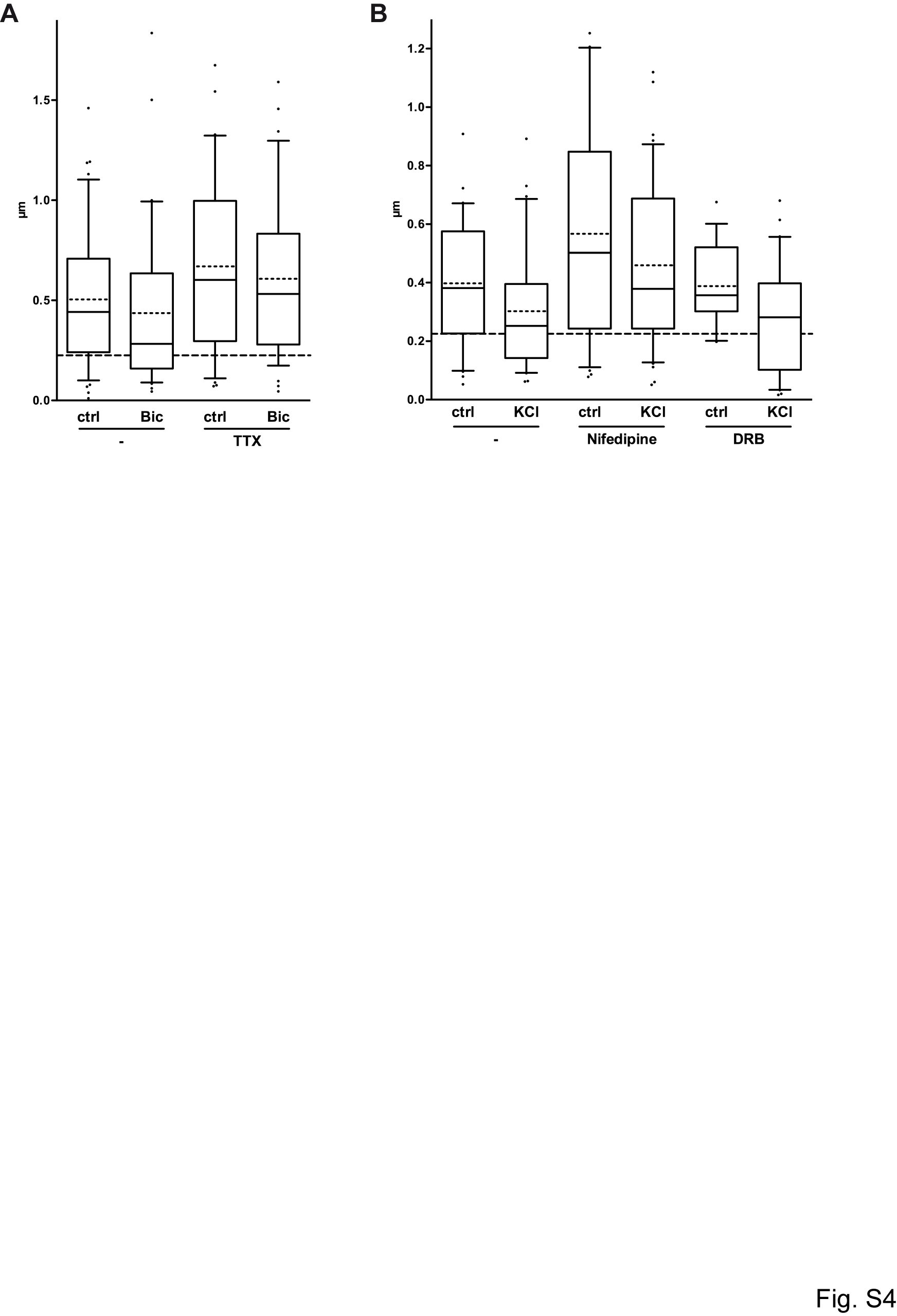

Supplement: Figure S4 — c-Fos relocation to TFs is depolarization-dependent. (A, B) Box and whiskers plot of the distribution of the distance between c-Fos locus and the nearest TF. Whiskers denote the 90th and 10th percentiles, box edges denote the 75th and 25th percentiles, solid lines denote medians, dashed lines denote averages. For each condition 30 to 41 FISH signals were analysed. (A) DIV10 cortical primary neurons were stimulated with 50 µM bicuculline for 45 minutes, either in the presence of 1 µM tetrodotoxin (TTX) or alone, and analysed by immuno-DNA FISH. (B) Cortical primary neurons were stimulated with 50 mM KCl for 45 minutes, either in the presence of 5 µM nifedipine, 50 µg/ml DRB or alone, and analysed by immuno-DNA FISH. (TIF) [file pgen.1003699.s005.tif]

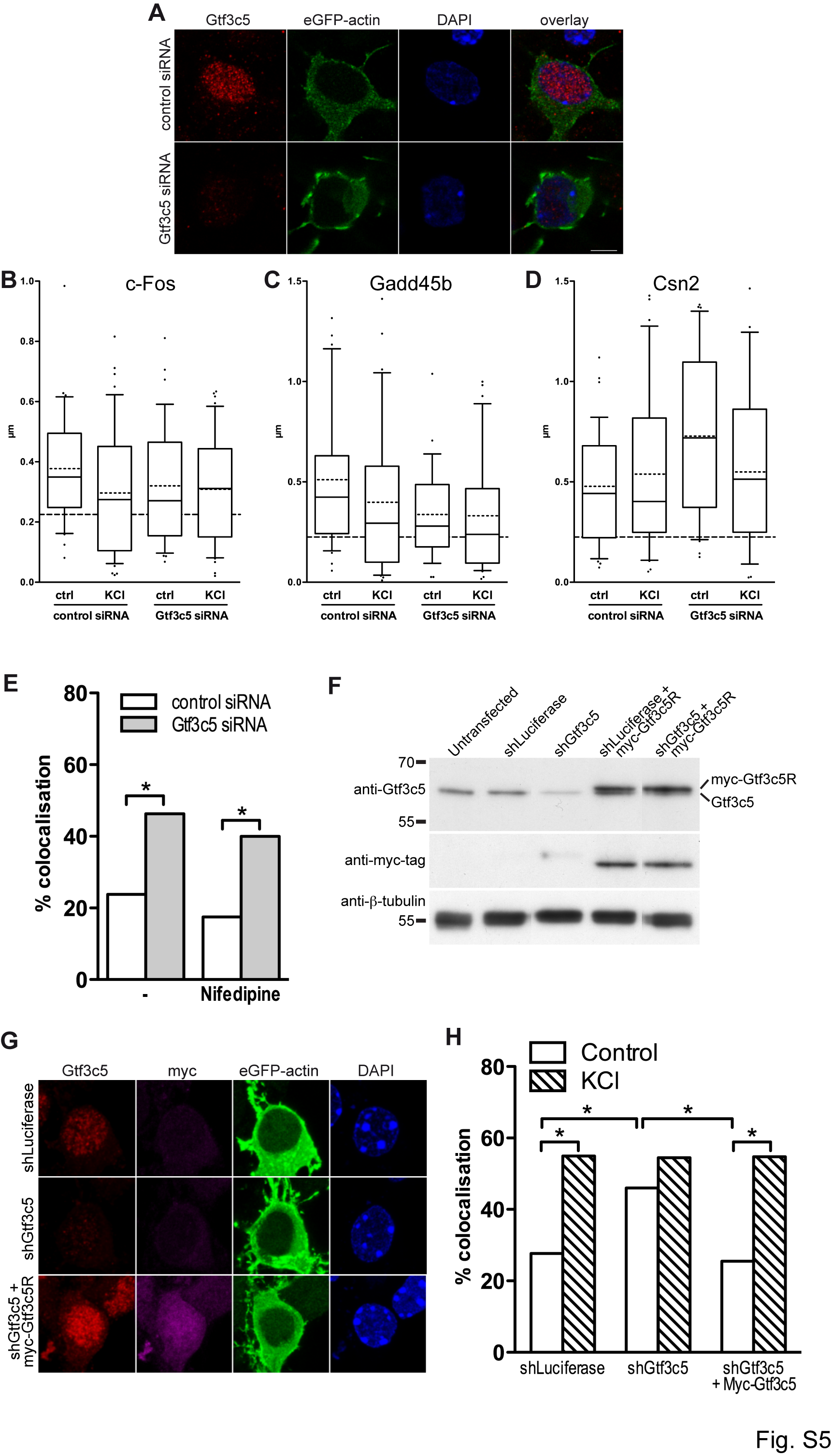

Supplement: Figure S5 — (A) Mouse cortical neurons were cultured for 2 days and transfected either with control or Gtf3c5 siRNA, in combination with an eGFP-actin expression vector. After three days, cells were fixed and subjected to Gtf3c5 and GFP immunofluorescence staining. Single confocal sections are shown (scale bar 5 µm). (B, C and D) Two days after plating, mouse primary cortical neurons were transfected with control or Gtf3c5 siRNA in combination with an eGFP-actin expression vector. After three days, neurons were stimulated with 50 mM KCl for 45 minutes or left untreated and analysed by immuno-DNA FISH targeting c-Fos, Gadd45b and Csn2 loci. Box and whiskers plots show the distribution of the distance between c-Fos (B), Gadd45b (C) and Csn2 (D) gene loci and the nearest TF. Whiskers denote the 90th and 10th percentiles, box edges denote the 75th and 25th percentiles, solid lines denote medians, dashed lines denote averages. For each condition 32 to 44 FISH signals were analysed. (E) c-Fos relocation to TFs induced by silencing of Gtf3c5 is not depolarization-dependent. Two days after plating, mouse primary cortical neurons were transfected with control or Gtf3c5 siRNA in combination with an eGFP-actin expression vector, and 5 µM nifedipine was added. After three days, neurons were stimulated with 50 mM KCl for 45 minutes or left untreated and analysed by immuno-DNA FISH targeting c-Fos locus. Culture in presence of nifedipine did not prevent the relocation of c-Fos to TFs, induced by Gtf3c5 silencing. Histograms show the percentage of co-localization with TFs of c-Fos gene locus (*, P<0.05, Fisher's exact test; n = 30 to 42 FISH signals per condition). (F) Western blot analysis of Gtf3c5 expression in NIH-3T3 cells transfected with pSUPER constructs expressing either short hairpin RNA targeting firefly Luciferase (shLuciferase, as a negative control), shRNA targeting mouse Gtf3c5 (shGtf3c5), shLuciferase and myc-tagged Gtf3c5 mutated to be shRNA resistant (myc-Gtf3c5R), or shGtf3 [file pgen.1003699.s006.tif]

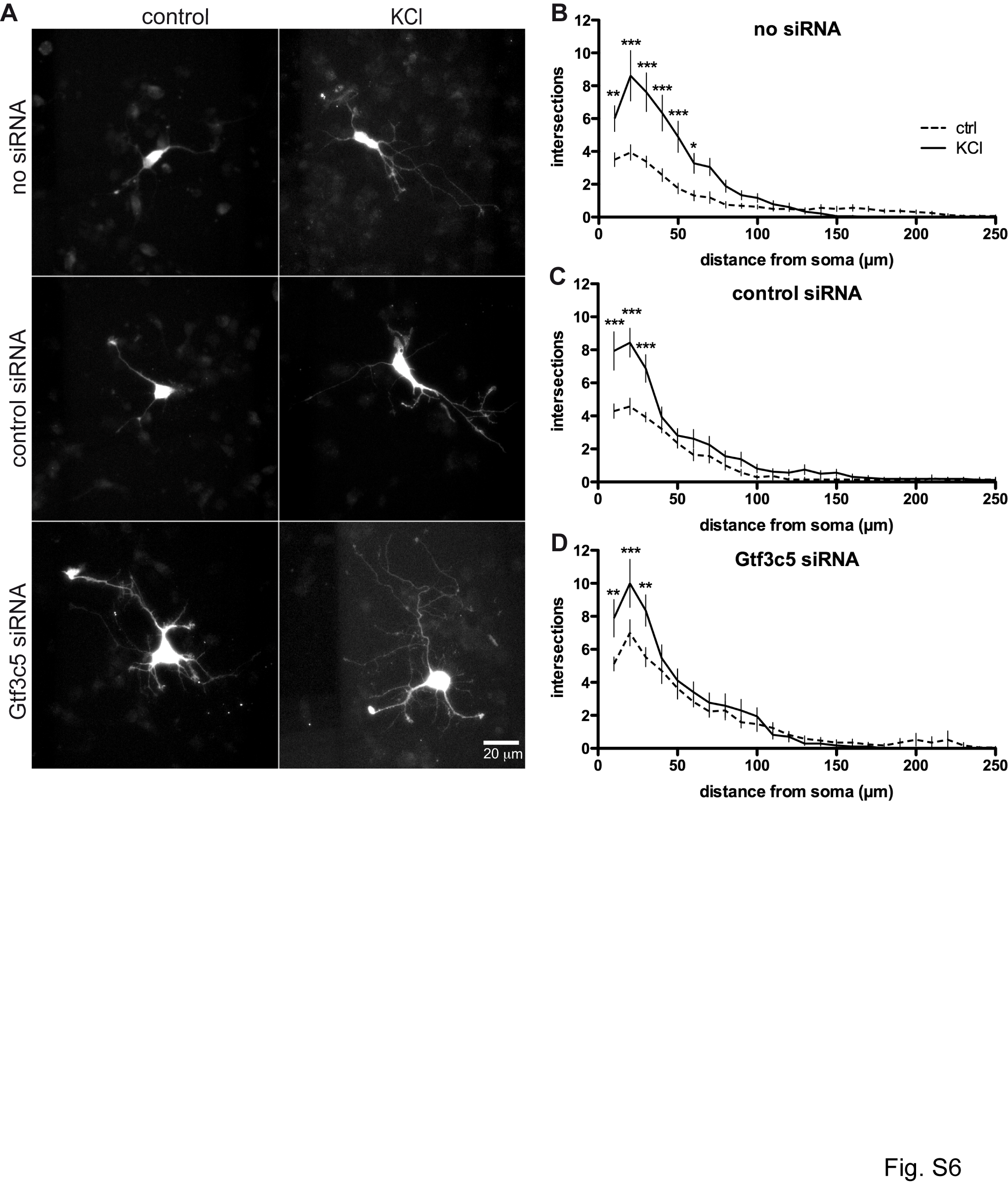

Supplement: Figure S6 — Sholl analysis of cortical neurons. (A) Original images of cortical neurons (shown in Figure 6A) transfected with a GFP-expressing vector alone or in combination with either control or Gtf3c5 siRNA. Neurons were cultured for 2 days in basal conditions or in presence of 50 mM KCl, followed by GFP immunostaining. Sholl profiles of neurons untransfected (B), transfected with a control siRNA (C) or with Gtf3c5 siRNA (D), and maintained for two days in basal conditions (dashed line) or exposed to KCl (solid line). For each distance point, the average number of intersections and s.e.m. are shown. At least 25 cells per condition were analysed (*, P<0.05; **, P<0.01; ***, P<0.001, two-way ANOVA). (TIF) [file pgen.1003699.s007.tif]
