# Supplementary material for: Binding of TFIIIC to SINE Elements Controls the Relocation of Activity-Dependent Neuronal Genes to Transcription Factories
Source: PLoS Genet. 2013 Aug 15;9(8):e1003699. doi: 10.1371/journal.pgen.1003699 (PMC3744447; doi:10.1371/journal.pgen.1003699)
Supplement: Table S1 — Summary of ChIPseq analysis. (DOC) [file pgen.1003699.s008.doc]

**Table S1**.

| Sample | Sequenced tags | Uniquely mapped tags | Percentage of uniquely mapped tags |
| --- | --- | --- | --- |
| Control | 6,870,576 | 3,470,045 | 50,5 % |
| NEE | 11,706,221 | 5,260,096 | 44,9 % |
