# Supplementary material for: Binding of TFIIIC to SINE Elements Controls the Relocation of Activity-Dependent Neuronal Genes to Transcription Factories
Source: PLoS Genet. 2013 Aug 15;9(8):e1003699. doi: 10.1371/journal.pgen.1003699 (PMC3744447; doi:10.1371/journal.pgen.1003699)
Supplement: Table S3 — Genomic coordinates of SINEs analysed. (DOC) [file pgen.1003699.s010.doc]

**Table S3**.

| SINE | coordinates |
| --- | --- |
| *c-Fos*^RSINE1^ | chr12:86,818,761-86,818,868 |
| *Gadd45b*^B1F^ | chr10:80,402,227-80,402,353 |
| *Jdp2*^B1^ | chr12:86,891,095-86,891,227 |
| *Gapdh*^B4^ | chr1:182,246,879-182,246,994 |
