# Supplementary material for: Binding of TFIIIC to SINE Elements Controls the Relocation of Activity-Dependent Neuronal Genes to Transcription Factories
Source: PLoS Genet. 2013 Aug 15;9(8):e1003699. doi: 10.1371/journal.pgen.1003699 (PMC3744447; doi:10.1371/journal.pgen.1003699)
Supplement: Table S4 — Primer sequences. (DOC) [file pgen.1003699.s011.doc]

**Table S****4**.

| Target sequence | Forward primer | Reverse primer |
| --- | --- | --- |
| c-Fos TSS | ggtcatgggacaacttccag | cagcgaaacacccttacaca |
| c-Fos^RSINE1^ | GGCTCTCCTGTCAACACACA | TCAGACAAGTCCTGGGTTCC |
| Gadd45b TSS | TTCCATCTCCAGCCAATCTC | ATCCCCCTCGAAATTAATCC |
| Gadd45b^B1F^ | tgtgtgtgtgtCCAGTCAGg | TAATCCCTGAGTGCCTTTGG |
| Arc TSS | gccctaccccagtgtctgta | acctacttcctgcccaacct |
| 5S rDNA | GGCCATACCACCCTGAACGC | CAGCACCCGGTATTCCCAGG |
| Jdp2^B1^ | gagggacaacctgagttcca | Gggcgacatgttctactcgt |
| Gapdh^B4^ | ttttccttcctccctcacct | Tcccatcctgtggatactgc |
| Control (Figure 2D) | gccctcactgcttctgagtc | Cccccatctttccctgtaat |
| Enh1 | AAAATCTGGCAGCCTTCTCA | AGCTGCAGAGAGACCCAAAA |
| Enh2 | GCAACAGGCTTCTGATTGGT | ACGTTGAGCACGTTTTCTCC |
| Enh3 | CAGGATGGGGTTCCTACTGA | GCACAGGCACAGATTTACCA |
| Enh4 | CAGGATTGAGCAGCTACACG | CTGTCACAGGCAGAGGTGAA |
| Enh5 | TCCCGCAATTCCTTTTACAG | ACCCACGGCTCCTTAGAGAC |
| c-Fos pre-mRNA | cgcagacgtcagggatattt | gagaagcattccggtcagag |
| c-Fos mRNA | GAATGGTGAAGACCGTGTCA | TGCAACGCAGACTTCTCATC |
| Gadd45b pre-mRNA | GCAACCCCAGTAACTTTGGA | CCTGCAGGAGAGAAGGAGTG |
| Gadd45b mRNA | gctgtggagtgtgactgcat | ggggtccacattcatcagtt |
| Arc mRNA | GGTGAGCTGAAGCCACAAAT | GCTGAGCTCTGCTCTTCTTCA |
| 5s rRNA | GGCCATACCACCCTGAACGC | CAGCACCCGGTATTCCCAGG |
| Gtf3c5 mRNA | ACGAGGCCAAGATGCTACAG | TGATGCCAATGATCTCCAAG |
| Gapdh mRNA | acccagaagactgtggatgg | cacattgggggtaggaacac |
